# Of buds and bits: a meta-QTL study identifies stable QTL for berry quality and yield traits in cranberry mapping populations (*Vaccinium macrocarpon* Ait.)

**DOI:** 10.3389/fpls.2024.1294570

**Published:** 2024-09-17

**Authors:** Andrew F. Maule, Jenyne Loarca, Luis Diaz-Garcia, Hector Lopez-Moreno, Jennifer Johnson-Cicalese, Nicholi Vorsa, Massimo Iorizzo, Jeffrey L. Neyhart, Juan E. Zalapa

**Affiliations:** ^1^ Department of Plant and Agroecosystem Sciences, University of Wisconsin-Madison, Madison, WI, United States; ^2^ Vegetable Crops Research Unit, United States Department of Agriculture - Agricultural Research Service, Madison, WI, United States; ^3^ Department of Viticulture and Enology, University of California, Davis, Davis, CA, United States; ^4^ P.E. Marucci Center for Blueberry and Cranberry Research and Extension Center, Rutgers University, Chatsworth, NJ, United States; ^5^ Department of Plant Biology, School of Environmental and Biological Sciences, Rutgers University, New Brunswick, NJ, United States; ^6^ Department of Horticultural Science, North Carolina State University, Raleigh, NC, United States; ^7^ Plants for Human Health Institute, North Carolina State University, Raleigh, NC, United States; ^8^ Genetic Improvement for Fruits & Vegetables Laboratory, United States Department of Agriculture-Agricultural Research Service, Chatsworth, NJ, United States

**Keywords:** American cranberry, QTL, meta-QTL, BLUP, phenotyping, perennial crops, fruit breeding

## Abstract

**Introduction:**

For nearly two centuries, cranberry (*Vaccinium macrocarpon* Ait.) breeders have improved fruit quality and yield by selecting traits on fruiting stems, termed “reproductive uprights.” Crop improvement is accelerating rapidly in contemporary breeding programs due to modern genetic tools and high-throughput phenotyping methods, improving selection efficiency and accuracy.

**Methods:**

We conducted genotypic evaluation on 29 primary traits encompassing fruit quality, yield, and chemical composition in two full-sib cranberry breeding populations—*CNJ02* (*n =* 168) and *CNJ04* (*n =* 67)—over 3 years. Genetic characterization was further performed on 11 secondary traits derived from these primary traits.

**Results:**

For *CNJ02*, 170 major quantitative trait loci (QTL; *R* ^2^ *≥* 0.10) were found with interval mapping, 150 major QTL were found with model mapping, and 9 QTL were found to be stable across multiple years. In *CNJ04*, 69 major QTL were found with interval mapping, 81 major QTL were found with model mapping, and 4 QTL were found to be stable across multiple years. Meta-QTL represent stable genomic regions consistent across multiple years, populations, studies, or traits. Seven multi-trait meta-QTL were found in *CNJ02*, one in *CNJ04*, and one in the combined analysis of both populations. A total of 22 meta-QTL were identified in cross-study, cross-population analysis using digital traits for berry shape and size (8 meta-QTL), digital images for berry color (2 meta-QTL), and three-study cross-analysis (12 meta-QTL).

**Discussion:**

Together, these meta-QTL anchor high-throughput fruit quality phenotyping techniques to traditional phenotyping methods, validating state-of-the-art methods in cranberry phenotyping that will improve breeding accuracy, efficiency, and genetic gain in this globally significant fruit crop.

## 1 Introduction

The “American” cranberry (*Vaccinium macrocarpon* Ait.) is part of a rich and diverse genus with over 500 species adapted to live in marginal habitats—thriving in acidic, peaty bogs, on the rims of sulfur-belching volcanoes, and as epiphytes in the upper story of forests (Vander Kloet, 1988; Vander Kloet and Avery, 2010). Prior to its breeding in the past two centuries, cranberry had been (and continues to be) a cultural, economic, and culinary facet of some indigenous peoples (especially those from modern-day “North America”) for several millennia. The development and advancement of *Vaccinium* germplasm for wide commercial use offers a chance to expand agriculture beyond fast-dwindling arable land, offering opportunities to expand nutritional diversity to the human diet.

Cranberry is one of the most important commercial species in the *Vaccinium* genus, along with blueberries, bilberries, and lingonberries (Chandler et al., 1947; Vorsa and Zalapa, 2019). In 2022, global yield of cranberries was around 600,000 metric tonnes, with the United States (USA) producing just over 60% of global yield, followed by Canada at around 35% and Chile (extrapolated) at just under 4% (FAOSTAT, 2022). The estimated raw production value of the USA 2022 cranberry harvest was around $304 million USD (USDA NASS, 2022), not considering value-added products (Alston et al., 2014). Despite its economic importance and nearly a century of breeding, most planted cultivars are limited to only one or two generations beyond wild germplasm, with only recent introduction of third-generation cultivars in managed marshes (Diaz-Garcia et al., 2020). Limited breeding progress stems from large planting space requirements, long establishment times (3–4 years), and extended evaluation times of 6–8 years before commercial release (Vorsa and Zalapa, 2019).

Cranberries exhibit unique plant architecture among woody fruit crops, with a growth habit that is characterized by low-growing vines producing short vertical lateral branches known as “uprights.” These uprights are classified into two types: vegetative uprights and reproductive uprights. Vegetative uprights develop apical buds with only vegetative meristems (vegetative buds) and thus only produce leaves. Reproductive uprights develop both vegetative and floral meristems (reproductive buds) and thus produce leaves while also sustaining the development of flowers and fruit. Apical bud induction and differentiation is determined by both management practices and by genetics (Bolivar-Medina et al., 2019). Cranberry yield traits, such as fruit size, quality, shape, and number, were traditionally measured on these flowering vines on a per-upright basis. As such, “reproductive upright traits” have been the traditional target of selection to phenotypically improve berry quality and yield. However, given the substantial cost and time required to accurately phenotype these traits, convention is transitioning toward measuring yield and chemistry on a plot-level (or per-unit-area) basis (Vorsa and Zalapa, 2019).

The past 7 years have shown incredible progress in cranberry molecular resource development and utilization (Covarrubias-Pazaran et al., 2018; Vorsa and Zalapa, 2019). In cranberry, advancement and cost-reduction in high-throughput genome sequencing technologies have enabled assembly of plastid and mitochondrial genomes (Fajardo et al., 2013; Diaz-Garcia et al., 2019, 2014), construction of two high-quality genome (Diaz-Garcia et al., 2021; Kawash et al., 2022), *de-novo* sequencing of cranberry transcriptomes (Georgi et al., 2013; Sun et al., 2015; Diaz-Garcia et al., 2021), linkage map development for agronomic traits (Georgi et al., 2013; Schlautman et al., 2015, 2017a; Covarrubias-Pazaran et al., 2016; Daverdin et al., 2017), and pilot applications in association mapping and genomic selection (Covarrubias-Pazaran et al., 2018; Diaz-Garcia et al., 2020; Neyhart et al., 2022).

These technologies have enabled several marker-trait association studies on an array of commercially important cranberry traits. For example, Georgi et al. (2013) found evidence of several quantitative trait loci (QTL) in four related populations for *field fruit-rot resistance*, *titratable acidity* (*TA*), f*ruit weight*, and *sound fruit yield* (*SFY*). Schlautman et al. (2015) constructed a high-density microsatellite linkage map in a breeding population and discovered QTL for mean fruit weight (*MFW*), total yield (*TY*), and biennial bearing index (*BBI*). Daverdin et al. (2017) generated high-density linkage maps in four diverse populations selected to demonstrate high levels of segregation for *field fruit-rot resistance* and found 15 QTL across all populations while demonstrating that yield traits segregate independently of *field fruit-rot resistance*. Image analysis and wet chemistry techniques have demonstrated the power to rapidly generate fruit shape, size, and color descriptors, which are important proxies for fruit quality and yield, and have identified QTL for anthocyanin production, *MFW*, and shape descriptors that are important for sweetened dried cranberry (SDC) production (Covarrubias-Pazaran et al., 2018; Diaz-Garcia et al., 2018a, 2018b). Finally, pilot applications in association mapping and genomic selection have been recently conducted in cranberry (Covarrubias-Pazaran et al., 2018; Diaz-Garcia et al., 2020; Neyhart et al., 2022).

Despite these significant advancements in molecular methods and genetic tools, no study has yet compared or validated trait phenotypes derived from traditional versus contemporary methods. We conducted QTL analysis on trait phenotypes obtained from contemporary phenotyping methods developed in the last three decades and on traditional phenotyping techniques that have been implemented by cranberry breeders in the last two centuries (Franklin et al., 1958; Eck, 1990; Vorsa and Zalapa, 2019). Many of the traits used in the current study are relevant to modern high-throughput phenomic tools, and others are traits traditionally collected per upright to make selection decisions (Diaz-Garcia et al., 2016). Leveraging advanced genomic and phenomic tools will accelerate genetic gain in cranberry breeding programs and improve understanding of which traditional traits are still useful and relevant in the modern context. This paper is the first to report correlations, heritabilities, and QTL based on both traditional and modern phenotyping methods. We propose that modern phenotyping methods are at least as accurate as traditional phenotyping methods, if not more so, for detecting heritable variation in reproductive upright traits, leading to identification of multi-year QTL for fruit yield and quality. We further expect that QTL identified across years and across studies (meta-QTL) will offer robust opportunities to perform marker-assisted selection for cranberry fruit quality and yield traits. Finally, validation of modern methods against traditional methods will open the doors to more efficient selection for these critically important traits.

## 2 Materials and methods

### 2.1 Plant material and traits collected

The two full-sib populations used in this study, *CNJ02* (*n =* 168) and *CNJ04* (*n =* 67), represent the most highly studied populations in the cranberry fruit yield and quality scientific literature (Schlautman et al., 2015, 2017b; Covarrubias-Pazaran et al., 2018; Diaz-Garcia et al., 2018b; Vorsa and Zalapa, 2019; Diaz Garcia et al., 2021). These populations were established in 2007 in 2.3 m^2^ field plots at Rutgers’ P.E. Marucci Center for Blueberry and Cranberry Research and Extension, Chatsworth, NJ (Vorsa and Johnson-Cicalese 2012), with experimental design described in previous studies (Schlautman et al., 2015; Covarrubias-Pazaran et al., 2016; Schlautman et al., 2017b; Diaz-Garcia et al., 2018b). *CNJ02* was derived from a cross between seed-bearing parent, *CNJ97_105_3* (cv. *Mullica Queen^®^*) and pollen-donating parent, *NJS98_23* (cv. *Crimson Queen^®^*); *CNJ04* was derived from reciprocal crosses between *CNJ97_105_3* (cv. *Mullica Queen^®^*) and cv. *Stevens*. Naming conventions of individual genotypes for populations *CNJ02* and *CNJ04* are as follows: *CNJ<YY>_<CN>_<GID>*, where *<YY>* is a two-digit designation indicating the year of the cross (2002 or 2004, respectively), *<CN>* is a number indicating the cross number (CN) for that year, *<GID>* is a genotype identifier (GID), and the underscore separates identifiers in the string. For example, *CNJ02_1_38* is a progeny from *CNJ02*, derived from the first cross (CN = 1) made in 2002, with genotype individual number 38 of that cross (GID = 38).

From 2011 to 2014, reproductive uprights were collected from field plots, 10 uprights per genotype. Traits from *CNJ02* and *CNJ04* each were recorded over 3 years, with *CNJ02* sampled for the years 2011–2013 and *CNJ04* sampled for 2011, 2012, and 2014. The traits measured largely comprise those traditionally considered to be commercially important in cranberries (Vorsa and Zalapa, *personal communication*), along with new fruit quality traits. Traits were loosely categorized into attributes measured directly, and traits features derived from other attributes. Traits measured directly include the categories *Upright Traits*, *Largest Berry Traits*, and *Plot Traits*; derived traits fall into the category *Berry Shape Chimera Parameters* calculated from berry shape composite representations (**Supplementary Table S1**). Traits will frequently be referred to by both their full names and acronyms to facilitate figure interpretation and cross-referencing with the manuscript. Trait acronyms will be defined once again in each new section or paragraph.

*Plot Traits* were sampled the same years as the uprights and are based on 0.09 m^2^ plot samples of fruit. These include *TY*, *SFY*, *mean fruit mass* (*MFM*), *percent fruit rot* (*PFR*), *total anthocyanins* (*Tacy*), *soluble solids* (*Brix*), *TA*, and *proanthocyanins* (*PACs*) (**Supplementary Table S1**) (Diaz-Garcia et al., 2018b).

*Upright Traits*, which include *Largest Berry Traits*, were recorded per upright, and their abbreviations are always prefixed with the letter “*U*” (**Supplementary Table S1**). *Upright Traits* include *total berry mass* (*UTBM*), *length* (*UL*), *secondary length* (*USL*), *dry leaf mass* (*UDM*), *rebud* (*URB*), *mean fruit mass* (*UMFM*), *number of pedicels* (*UNPs*), *number of pedicels without berries* (*UN0*), *number of pedicels with mature berries* (*UNBs*), *number of pedicels with aborted flowers* (*UNAFs*), and *number of pedicels with aborted berries* (*UNABs*).

*Largest Berry Traits* were measured on the largest berry per upright (10 berries per genotype). *Largest Berry* yield traits include *berry length* (*UBL*), *berry width* (*UBW), berry length:width ratio* (*ULvW*), and *berry mass* (*UBM*). *Largest Berry* quality traits include *berry shape* (*UBS)*, *number of seeds* (*UNSs*), *calyx diameter* (*UCD*), *calyx lobe fold pattern* (*UCLP)*, *calyx lobe size* (*UCLS)*, *calyx end shape* (*UCES)*, *berry pedicel end shape* (UBES), and *berry bloom level* (*UBBL*)*—*a measure of berry epicuticular wax levels.

**Figure 1:**
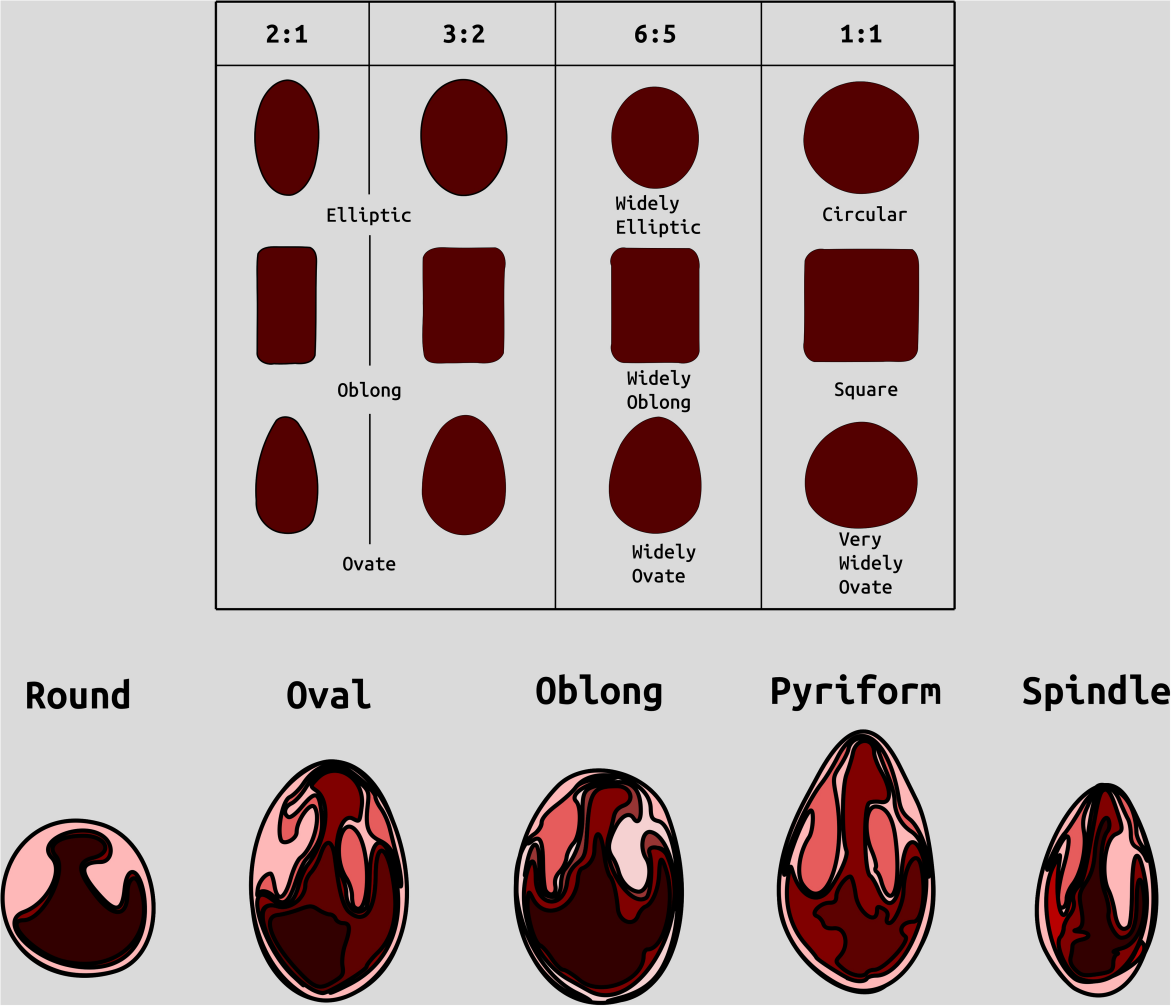
Examples of categories of berry shape parameters used to help classify cranberry shape. Modified from (Franklin et al. 1958).

**Figure 2:**
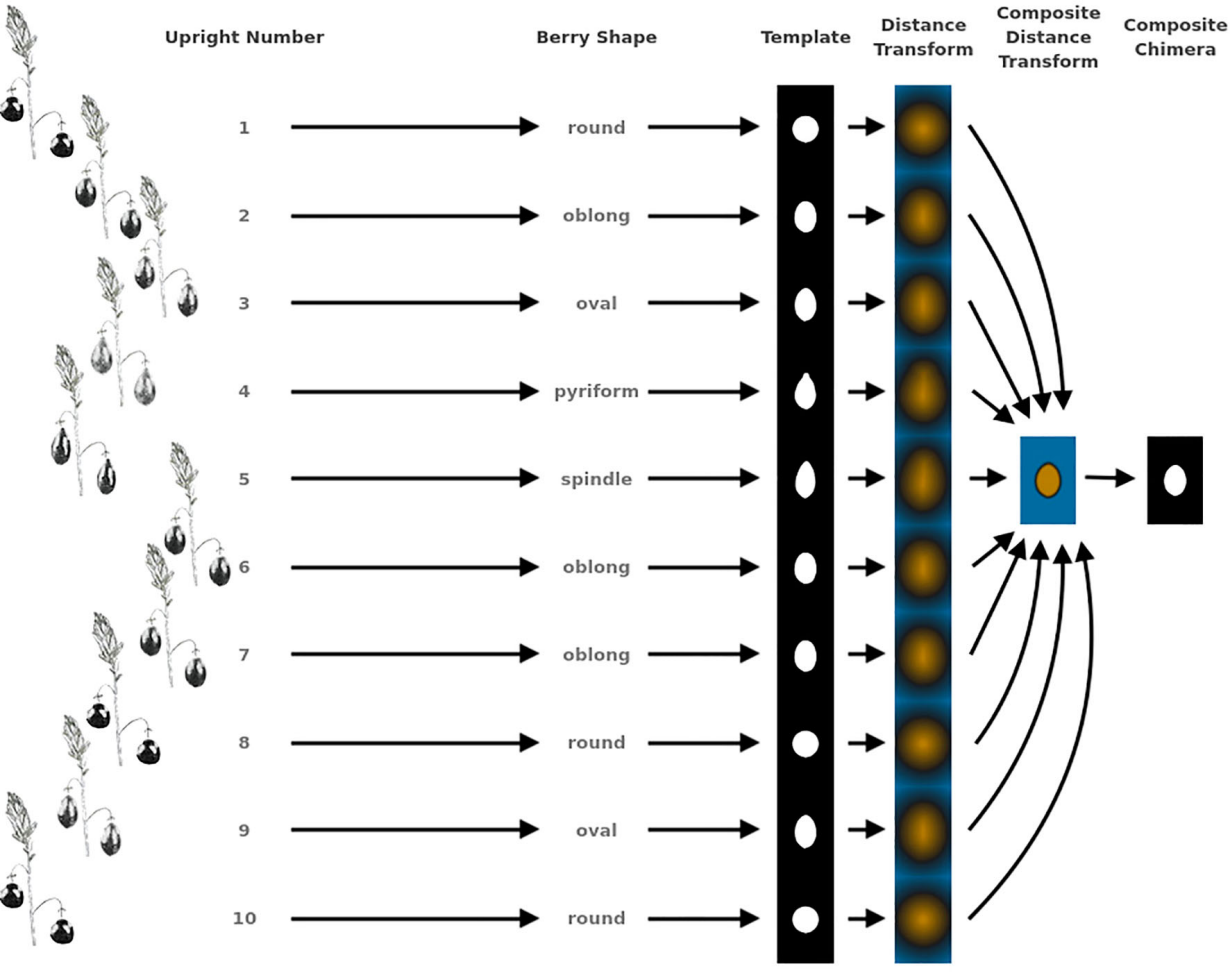
Example showing the methodology for generating representative genotype shape, or berry chimera, from 10 upright samples. This berry chimera is subsequently used as an image to generate digital image processing shape descriptors used in creating quantifying features for QTL mapping.

*Berry Shape Chimera Properties* are derived from the composite representations, or chimeras, of five berry shape categories (**Figure 1**). The berry chimera is rendered from the average shape of the 10 largest berries (**Figure 2**). These parameters were extracted and mapped to provide a data integrity benchmark of the subjective, categorical berry shape traits against their per-upright berry trait analogs (**Supplementary Tables S1** and section 2.2).

### 2.2 Trait evaluation and transformation

All categorical traits were transformed to numeric, discrete values (except for largest berry shape). Higher values represent more favorable characteristics. **Supplementary Table S3** displays the conversions of categorical traits to numeric values.

Largest berry shape was quantified by replacing scored shape categories with their canonical digital image shapes using the template images provided to trait evaluators (**Figure 1**). Digital image shape descriptors derived from the representative berry chimera are listed in **Supplementary Table S1** under category *Berry Shape Chimera Parameters*. These images were initially converted to binary images, normalized by centering all representations, and scaling so that all shape templates have the same area. Thereafter, a chimeric berry representation was derived for each genotype year by combining and thresholding the differential distance transformed representations of each corresponding upright’s normalized berry shape (**Figure 2**). A distance transform representation of a binary image is the Euclidean distance of any foreground pixel to its nearest background pixel. The differential distance transformation is the difference between the distance transformation of the normalized binary berry template image and the same image inverted. Positive values indicate foreground pixels, with higher values indicating the centroid of binary images. By combining the differential distance transforms and thresholding on positive values, a chimeric binary berry image representation per genotype year can be generated. The berry chimera therefore represents a synthesized berry image derived from the composition of multiple categorical berry shapes (10 berry shape classes per genotype). Shape descriptors are calculated from the synthesized berry chimera, allowing for quantitative analysis of shape traits for mapping. Traits *upright chimera unsigned manhattan chain code–X-axis* (*UKUX)* and *upright chimera unsigned manhattan chain code–Y-axis* (*UKUY)* are log 10  derivations of unsigned manhattan chain codes (UMCC) (Žalik et al., 2016). A chain code is a numerical representation describing the contour path of an object. UMCCs are one of many chain codes used to describe contour shapes and were chosen here for their highly compressible representation. Other chimera shape descriptors outlined in **Supplementary Table S1** include *chimera shape eccentricity* (*UKEC*), *chimera length:width ratio* (*UKLvW*), *chimera tortuosity* (*UKTO*), and *chimera solidity* (UKSO). *Eccentricity* (*EC*) of a closed contour is a mathematical descriptor for the curvature of an ellipse, with zero indicating a perfect circle and values closer to one signifying a higher length:width ratio. Tortuosity describes the *“waviness”* of an object’s contour and, in this case, a slope chain code (SCC) method was applied to generate this tortuosity value (Bribiesca, 2013). Solidity describes the shape density relative to its convex hull. Lower solidity values indicate more *waviness* in the berry contour, while higher values have smoother contour curvature.

All traits were curated by removing entries marked as rotten, and outliers were detected and trimmed with the outlierTest() function of the car package (RRID: SCR_022137) with a default cutoff of *p <* 0.05 using a linear model of the trait regressed on population, genotype, and year (Fox and Weisberg, 2019). Additional observations were culled if they exceeded three standard deviation units from the mean under a Gaussian standard distribution. Subsequent analysis and trait mapping were applied to all traits based on the mean trait values across the ten sampled uprights, for each genotype year. From these upright means, Pearson correlation coefficients were calculated for all traits and averaged across months and all sampled years. Correlation heatmap plots were generated using the corrplot package (RRID: SCR_023081), with statistical *p* values calculated using the ggcorrplot package (Kassambara, 2019; Wei and Simko, 2021). Trait correlations were partitioned using hierarchical agglomerative clustering cut at an absolute value correlation tree height equal to 0.6. These partitions form clusters, or cliques, of traits, delineated by heavy black lines in the generated heatmaps. Only non-singleton clusters are reported in the results.

### 2.3 Linkage maps

Linkage maps used in this study were previously created from a combination of robust single sequence repeat (SSR) markers and genotype-by-sequencing (GBS) single-nucleotide polymorphic (SNP) markers (Schlautman et al., 2017b). SSR marker data generated for *CNJ02* (541 SSRs) and *GRYG* (189 SSRs) were discovered and curated as described in Schlautman et al. (2015) and Covarrubias-Pazaran et al. (2016). For GBS markers, genomic DNA was extracted from flash frozen leaf tissue and EcoT221-digested DNA fragments were uniquely barcoded for all progeny and parents of *CNJ02* and *CNJ04* using the approach described by Elshire et al. (2011). These fragments were sequenced (single-end) at the Cornell University Biotechnology Resource Center Genomics Facility on a Illumina HiSeq 2000 platform. A reference Tassel GBS analysis pipeline (Bradbury et al., 2007) was used to filter and process the resulting sequence reads and call SNPs in the resulting datasets for the *CNJ02* and *CNJ04* populations using the parameters described in Schlautman et al. (2017b) and aligned to the cranberry reference genome produced by Polashock et al. (2014). SNPs with >20% missing data, minor allele frequency (MAF) of <10%, or severely distorted segregation ratios were removed. Linkage analysis on the filtered SSR and SNP markers was performed with JoinMap v4.1, using a pseudo-testcross method, and biparental consensus linkage maps were separately generated for *CNJ02* (*n =* 3925) and *CNJ04* (*n =* 3081) (Schlautman et al., 2017b). Additionally, a composite linkage map (*n =* 1560) derived from three cranberry populations, *CNJ02*, *CNJ04*, and *GRYG*, was generated using a linear programming approach as described by Schlautman et al. (2017), and is available on the Genome Database for Vaccinium site under the map identifier Cranberry-Composite_map-F1 (https://www.vaccinium.org/bio_data/1070). QTL generated herein used the composite linkage map in order to facilitate cross-population comparison. Population-specific biparental consensus linkage maps were used to produce the additive genomic relationship matrices described in Equation 2.1.

### 2.4 Estimating breeding values and heritability

Equation 2.1 shows the mixed model used in estimating best linear unbiased predictors (BLUPs) within years (Henderson, 1975). The equation variables are defined as follows: y = phenotype value, μ = intercept (global mean of trait), $Z_{g}$ = genotype random-effect incidence matrix, g = genotypic effects (BLUPs), $Z_{r}$ = row random effect incidence matrix, r = row effect, $Z_{c}$ = column random effect incidence matrix, c = column effect, $Z_{s}$ = 2D-spline random effect incidence matrix, s = spline effect, ϵ = residuals, G = genotype variance-covariance matrix (De Los Campos et al., 2015), A = additive genomic relationship matrix (Endelman, 2011), $\sigma_{a}^{2}$ = additive genomic variance, I = identity matrix, $\sigma_{r}^{2}$ = row variance, $\sigma_{c}^{2}$ = column variance, $\sigma_{s}^{2}$ = 2D-spline variance, $\sigma_{\epsilon }^{2}$ = residual error variance.


2.1 $$y=\mu +Z_{g}g+Z_{r}r+Z_{c}c+Z_{s}s+\epsilon$$

*where* g∼𝒩(0,G) , $G=A\sigma_{a}^{2}$ , $r∼\text{𝒩}(0,I\sigma_{r}^{2})$ , $c∼\text{𝒩}(0,I\sigma_{c}^{2})$ , $s∼\text{𝒩}(0,I\sigma_{s}^{2})$ , *and* $\epsilon ∼\text{𝒩}(0,I\sigma_{\epsilon }^{2})$ .

Equation 2.2 displays the across year mixed model used in estimating BLUPs. Year is modeled as a fixed effect. All symbols are the same as in Equation 2.1 but with the additional term $X_{e}$ = fixed-effect year incidence matrix, e = year effect, $Z_{ge}$ = genotype-by-year random-effect incidence matrix, ge = genotype-by-year effect, and $\sigma_{ge}$ = genotype-by-year variance.


2.2 $$y=\mu +X_{e}e+Z_{g}g+Z_{ge}ge+Z_{r}r+Z_{c}c+Z_{s}s+\epsilon$$

where levels of $X_{e}\in \{2011,2012,2013,2014\}$ , and $g_{e}∼\text{𝒩}(0,\sigma_{ge}^{2})$ .

The mixed models detailed in Equations 2.1 and Equation 2.2 were fit using utilities defined in the R package (R Core Team, 2024) sommer (Covarrubias-Pazaran, 2016). The additive genomic relationship matrix *A* (also known as additive relationship matrix) is a variance-covariance matrix that was constructed using the sommer function A.mat() on biallelic markers (SNP) from each population’s respective biparental consensus linkage map. Modeling the genetic relatedness between genotypes with the *A* matrix adjusts for differences absent a structured experimental design. Spatial effects were also compensated for by modeling this variation as 2D-spline random effects and as row and column random effects. The 2D-spline effects are continuous random variables that model spatial variation that does not track first-order polynomial (straight-line) field effects or does not trace along the row and column effects. The row and column random effects models spatial variation by blocking according to the predefined row and column indices of the plots. The BLUPs [also known as genomic estimated breeding values (GEBV)], were estimated using the mmer() function of the sommer package.

Model selection was performed from a full model search on the random terms of models defined in Equation 2.1 or Equation 2.2 using the *Akaike Information Criteria* (AIC) (Akaike, 1974). The model with the lowest AIC was subsequently used to estimate BLUPs, balancing model complexity with parsimony. For across-year mixed model BLUP estimates, the genotype-by-year interaction effect was excluded from the model fit if it did not display significance (*p <* 0.05) using a likelihood ratio test. Random term variance estimates were used to calculate additive genomic heritability ( $h^{2}$ ) of each trait (De Los Campos et al., 2015).

Equation 2.3 displays the formula for calculating within-year narrow-sense genomic heritability, using variances estimated from fitting the mixed model (Equation 2.1). Across year genomic heritability is calculated as shown in Equation 2.4, using variances estimated from the mixed model (Equation 2.2), with n representing the number of distinct years fit per trait.


2.3 $$h^{2}=\frac{\sigma_{a}^{2}}{\sigma_{a}^{2}+\sigma_{\epsilon }^{2}}$$

2.4 $$h^{2}=\frac{\sigma_{a}^{2}}{\sigma_{a}^{2}+\frac{\sigma_{ge}^{2}}{n}+\frac{\sigma_{\epsilon }^{2}}{n}}$$

### 2.5 QTL mapping

QTL mapping was performed by using the CRAN (RRID: SCR_003005) package r/QTL (RRID: SCR_009085), a software toolkit for mapping experimental crosses (Broman et al., 2003). To infer QTLs, previously modeled genotype BLUPs were substituted in lieu of raw phenotypes in the R/qtl cross table. QTL were detected using two methods: a single-QTL interval mapping method and a model selection approach. Both methods used Haley-Knott regression to model QTL between genetic map markers (Haley and Knott, 1992). The single-QTL method uses the scanone() function, with significant QTL determined using scanone() run against 1,000 permutations of the phenotypes in order to simulate the log of odds (LOD) distribution of the NULL model. The model selection approach uses the stepwiseqtl() function, which runs a forward/backward model search algorithm by which additive and interacting terms are successively added to the model, followed by “backward” pruning of other model terms that optimizes a penalized LOD score. The penalized LOD score uses $n^{th}$ percentile thresholds derived from running scantwo() against 1,000 permutations of the phenotypes in order to control the false positive rate at n percent (Broman et al., 2009). More complex models are penalized higher to reduce model overfitting. For single-QTL interval mapping, the significance threshold was set for QTL with LOD scores above the $80^{th}$ permutation percentile, while for model selection, penalized LOD scores were derived from thresholds determined from the $95^{th}$ permutation percentile. QTL inferred through statistical association mapping approaches are subsequently referred to as principal or primary QTL.

### 2.6 Meta-QTL

**Figure 3:**
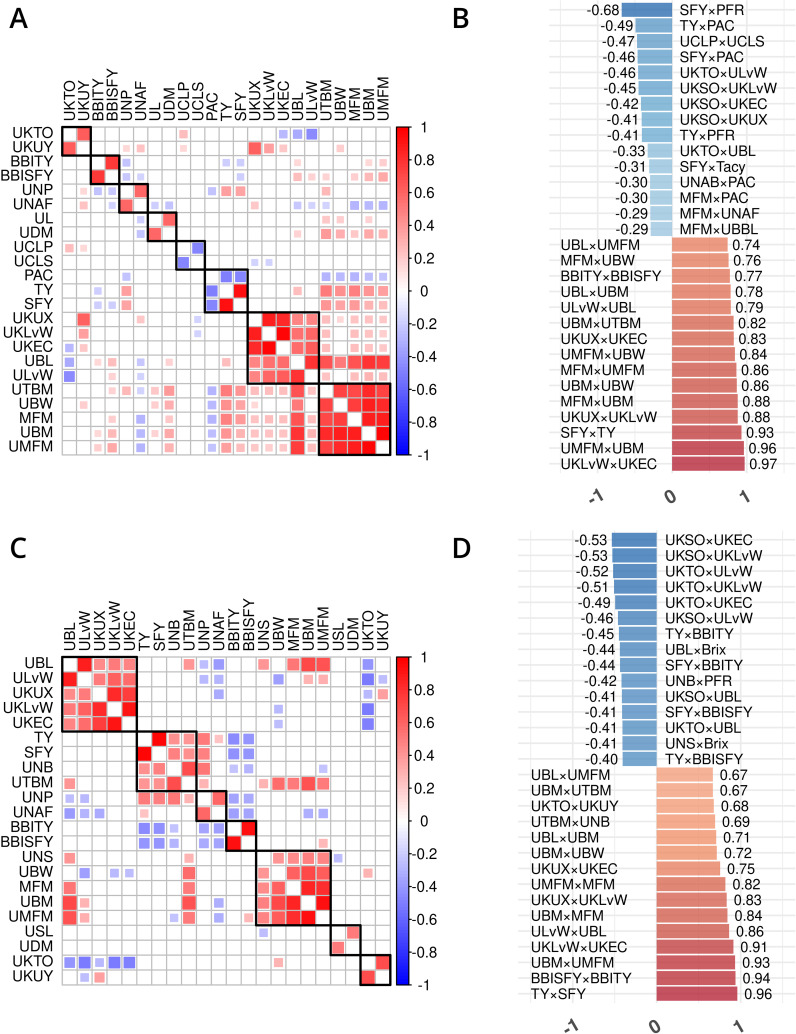
Trait phenotype correlation heatmaps for traits in cranberry populations *CNJ02* **(A, B)** and *CNJ04* **(C, D)**. Only traits found within non-singleton hierarchical clusters are shown in **(A, C)**, where black squares delineate clusters. The 15 largest positive and 15 smallest negative correlations are shown in **(B,D)**. Only significant pairwise trait correlations (*p* < 0.05) are displayed.

Co-located QTL, or meta-QTL, represent collections of principal marker associations that are stable across corresponding traits, years (multiple models), populations, QTL inference methods, and/or other studies. Major QTL are defined as those QTL with a mean percent marker variance explained (marker $\bar{R^{2}}$ ) greater than or equal to 10%. Within-trait major QTL found for populations *CNJ02* and CNJ04 were assembled from principal QTL that were stable in at least three of the four models (single-year models on three separate years as defined in Equation 2.1 and the multi-year model defined in Equation 2.2). To construct multi-trait composite meta-QTL, traits were grouped into correlated sets based on observed trait Pearson correlation blocks (**Figure 3**) for this study, or similar groups of traits across studies. Multi-trait composite meta-QTL were grouped according to the categories detailed in **Tables 1A**, **1B**, **Supplementary Tables 1A, 1B**. Within-trait major meta-QTL were assigned a separate group per trait. Using the lower and upper LOD 1.5 interval extents, all pairwise principal QTL within the same group were represented in an undirected graph structure, where nodes constitute the primary QTL and edges indicate QTL–QTL overlap. A graph was assembled for each set and trait group using the Python library NetworkX, and composite meta-QTL were synthesized from a maximal clique approximation algorithm, where each maximal clique represents a new meta-QTL (Van Rossum, 2007; Hagberg et al., 2008). For each maximal clique, the new left and right extents of the meta-QTL were taken from the maximal LOD 1.5 left extent and the minimal LOD 1.5 right extent. The meta-QTL’s synthetic position was calculated as the center, or mean, of its extents. Additionally, information was recorded for each meta-QTL by compiling summary statistics such as population count, study count, year count, mapping method count, trait count, and mean marker variance explained by QTL. Other meta-QTL information compiled included the composite list of traits, populations, models (temporal consistency), and studies.

**Table 1A:** Trait groupings for each set of co-located QTL in present study.

Trait Groups^a^	Filter^b^
***CNJ02***	$\bar{R^{2}}$ ≥ 10%
* mean fruit mass (MFM, UMFM); berry width (UBW);* * berry mass (UBM); total berry mass (UTBM)*	*Year count* ≥ 4
* total yield (TY); sound fruit yield (SFY); proanthocyanins (PAC)*	
* berry length (UBL); chimera eccentricity (UKEC);* * berry length:width (ULvW), chimera length:width UKLvW);*	
* upright length (UL); upright dry leaf mass (UDM)*	
* number of pedicels (UNP); number of pedicels with aborted flowers (UNAF)*	
***CNJ04***	$\bar{R^{2}}$ ≥ 10%
* UNP; UNAF*	*Year count* ≥ 4
* upright secondary length (USL); UDM*	
* number of seeds (UNS); UBW, MFM, UMFM; UBM*	
* number of pedicels with mature berries (UNB); TY; SFY; UTBM*	
* UBL; ULvW, UKLvW; UKEC*	
***CNJ0x***	$\bar{R^{2}}$ ≥ 10%
* UNP; UNAF*	*Year count* ≥ 4
* MFM, UMFM; UBM; UBW;*	*Populations* ≥ 2
* TY; SFY*	
* UBL; ULvW, UKLvW; UKEC*	

^a^Bold entries designate the separate sets used in co-location QTL analysis. ^b^Multiple filter rules are combined with a logical AND operation. Trait groupings for each set of co-located QTL in present study. Trait groups were selected based on correlated clusters or similar categories of traits. The filter column details the constraints applied to the synthetic meta-QTL that are displayed in the linkage maps. $\bar{R^{2}}$ is the mean percent marker variance explained across all composite QTL. Trait acronyms are provided upon first use in table and are abbreviated thereafter.

**Table 1B:** Trait groupings for each set of co-located quantitative trait loci (QTL) across cranberry studies (present study included).

Trait groups^a^	Populations	Filter^b^
***CNJ0x & Diaz-Garcia 2018a***	*CNJ02*, *CNJ04*, *GRYG*	$\bar{R^{2}}\geq 10\%$
*MFM, UMFM, UBM, UTBM, BW, UBW, UBL, BL, berry area (BA)*		Trait count≥3
* TY, SFY, PAC*		Study count≥2
* EC, UKEC, LvW, ULvW*		
***CNJ0x & Diaz-Garcia 2018b***	*CNJ02*, *CNJ04*, & *GRYG*	$\bar{R^{2}}\geq 10\%$
*MFM, UMFM, UTBM, UBW, UBM, UBL*		Populations≥2
* TY, SFY, PAC*		Study count≥2
*Berry color (BCOLOR), berry color variance (BCOLORVAR)* *total anthocyanin (Tacy)*, *total anthocyanin in September (TACY_SEP)*, *total anthocyanin in October (TACY_OCT)*, *difference in Tacy between Sept. and Oct. (TACY_DIFF)*		
***CNJ0x & Schlautman 2015***	*CNJ02*, *CNJ04*	$\bar{R^{2}}$ ≥10%
*MFM, UMFM, UTBM, UBM, UBL, UBW*		Study count≥2
*TY, SFY, PAC*		
***CNJ0x & Schlautman 2015 & Diaz-Garcia 2018a***	*CNJ02*, *CNJ04*, *GRYG*	$\bar{R^{2}}\geq 10\%$
* MFM, UMFM, UTBM, UBM, BL, UBL, BW,UBW, BA*		Trait count≥3
* TY, SFY, PAC*		Study count≥2
*EC, UKEC, LvW, ULvW*		

a Bold entries designate the separate sets used in co-location QTL analysis.

b Multiple filter rules are combined with a logical AND operation. Trait groupings for each set of co-located quantitative trait loci (QTL) across cranberry studies (present study included). Trait groups were selected based on correlated clusters or similar categories of traits. The filter column details the constraints applied to the synthetic meta-QTL that are displayed in the linkage maps. $\bar{R^{2}}$ is the mean percent marker variance explained across all composite QTL. Trait acronyms are provided upon first use in table and are abbreviated thereafter.

**Figure 4:**
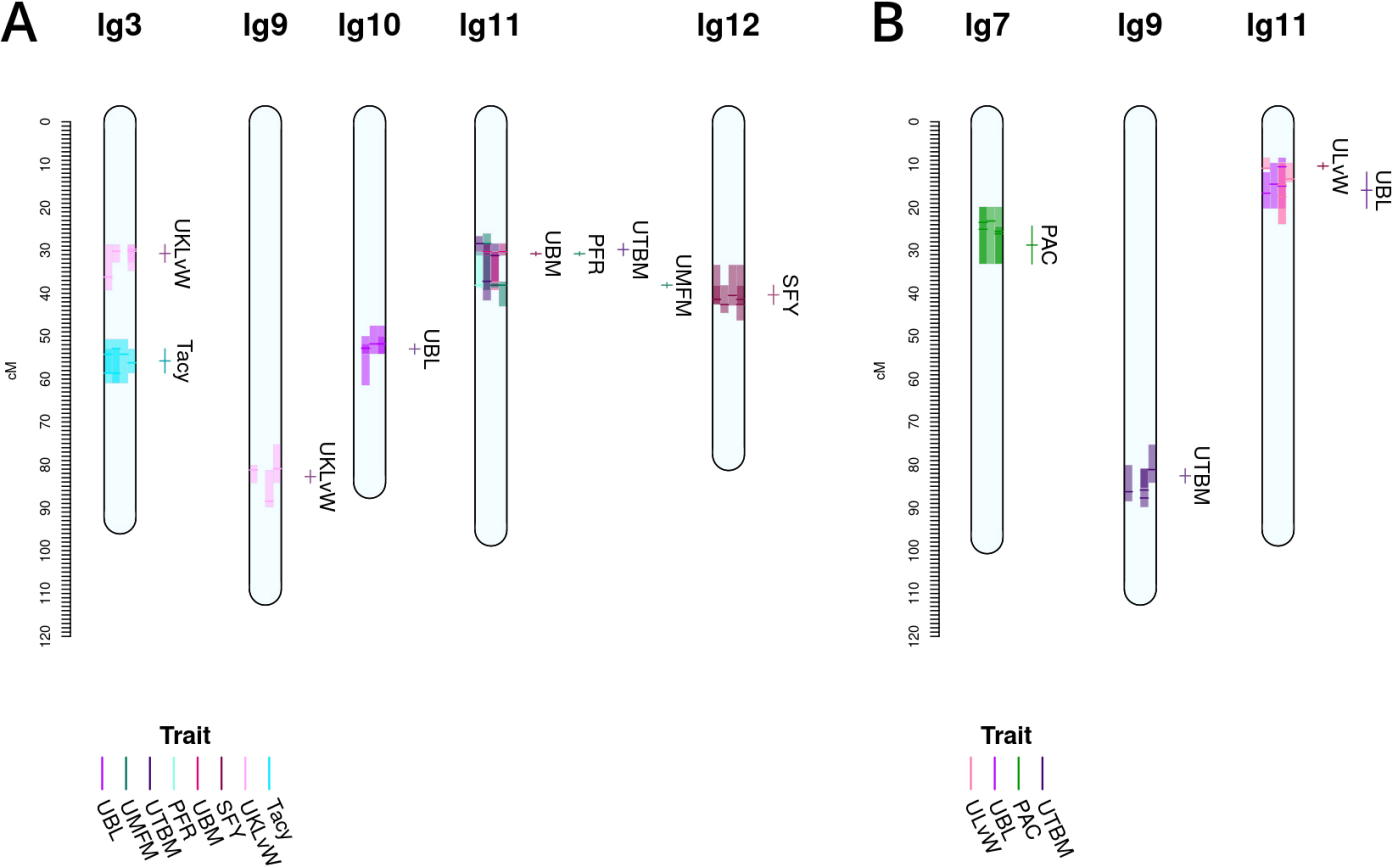
Co-located, stable QTL in cranberry populations *CNJ02* **(A)** and *CNJ04* **(B)** found in at least three of four BLUP models. Only QTL with $\bar{R^{2}}\geq 10\%$ are shown. Shaded bars represent the 1.5LOD confidence interval for the original QTL, with the darker line segments indicating the location of the QTL. The linkage groups are divided into four columns, one for each fitted BLUP model—from left to right: 2011, 2012, 2013, and *all years* **(A)**; and 2011, 2012, 2014, and *all years* **(B)**. Each QTL interval is color-coded by its corresponding trait. The labels and interval lines to the right of linkage groups show the stable meta-QTL associated with their respective genomic regions.

Genomic plots of composite meta-QTL were generated using a customized version of the CRAN package LinkageMapView (Ouellette et al., 2018). For each multi-trait set annotated in **Tables 1A**, **1B**, a representative linkage map of selected meta-QTL was generated. Line segments represent the maximal LOD position of the principal trait QTL, linkage map fills represent the original LOD 1.5 intervals, labels identify the respective traits of the composite, and interval bars demarcate the intersection of principal QTL 1.5 LOD intervals. Traits and intervals are colored as defined in each figure’s respective legend (**Figures 4** **–** **6**). Within-trait meta-QTL for *CNJ02* and *CNJ04* are graphed similarly to multi-trait meta-QTL, but with each linkage group subdivided into four columns delineating the year models (**Figure 4**).

## 3 Results

### 3.1 Correlations

Prominent trait correlations illuminate phenotypic relationships for population *CNJ02* (A and B) and *CNJ04* (C and D) (*p <* 0.05) (**Figure 3**). The bar plots in **Figures 3B, D** show the 15 top positive and 15 bottom negative inter-trait correlations per respective population. Only significant correlations (*p <* 0.05) are displayed in **Figures 3B, D**. Correlated trait clusters in *CNJ02* include {*PACs*, *TY*, *SFY*}, {*MFM*, *UMFM*, *UBM*, *UTBM*}, and {*UCLP*, *UCLS*}, {*UKTO*, *UKUY*}, {*biennial bearing index-total yield* (*BBITY*), *biennial bearing index-sound fruit yield* (*BBISFY*)}, {*UNAFs*}, {*UBL*, *UDM*}, and {*UBL*, *UKLvW*, *UKEC*, *ULvW*, *UKUX*} (**Figure 3A**). Pairwise positive associations evident in the *CNJ02* dataset include {*SFY × TY*}, {*MFM × UBM*}, {*MFM × UMFM*}, {*UBM × UTBM*}, and {*MFM × UBW*}. Negative pairwise correlations found in the *CNJ02* dataset include {*TY × PAC*}, {*UCLP × UCLS*}, {*SFY × PAC*}, {*SFY × Tacy*}, {*PAC × UNABs*}, {*MFM × PAC*}, {*MFM × UNAFs*}, and {*MFM × UBBL*}. For *CNJ04*, correlation clusters include the {*TY, SFY*, *UTBM*, *UNBs*} cluster and the {*MFM*, *UMFM*, *UBM*} cluster (**Figure 3C**).

### 3.2 Heritabilities

Generally, *all-year* model trait BLUPs (Equation 2.2) have larger heritabilities relative to their respective fitted model trait BLUPs for individual years. Traits with consistently high heritability in both populations are displayed in **Figure 7**. Heritability values are calculated from the inter-population mean of the *all-year* model heritability (**Figure 7A**), and separately, the within-year model heritability averages between *CNJ02* and *CNJ04* (**Figure 7B**). *ULvW* has remarkably high heritability in both populations, followed by *UBL* and *UNSs*. *Tacy*, a desirable trait associated with fruit color, also shows evidence of high heritability. High heritabilities in *UBM*, *UMFM*, and *TY* indicate strong potential for selecting for higher yields in both populations. The fruit rot *SFY* and *PFR* exhibited low to modest heritabilities (0.3 ≤ *h* ^2^ ≤ 0.6) in both populations. Fruit quality traits such as *TA* and *Brix* exhibit very low *all-year* heritabilities in population *CNJ02* but higher heritability in *CNJ04* (**Figure 8**).

### 3.3 Breeding value estimates

Population *CNJ02* and *CNJ04* both displayed evidence of transgressive segregation for many of the traits. **Tables 2**, **3** show summary statistics for traits and their associated BLUPs for the *all-year* model. Rows are sorted by decreasing heritability, and only display the top 50% of models based on heritability ( $h^{2}\;\geq \;0.47$ ) and with significant genotypic effects (*p <* 0.05). Raw trait associated statistics are subscripted with the italicized letter *r*, and BLUP associated statistics are subscripted with the italicized letter *b*. The top and bottom five representative genotypes are also presented both for recorded traits and for genotype BLUPs.

**Table 2:** Cranberry population CNJ02 trait and BLUP summary for all-year model with heritability above 0.47. Only models with significant (*p* < 0.05) genotypic effects are displayed.

Trait	h^2*^	F_1_ progeny					Parents				
		Min_r_^†^	Min_b_^‡^	μ_r_ ± SE^§^	μ_b_ ± SE^#^	Max_r_^⚭^	Max_b_^⚔^	P_1r_^♠^	P_1b_^♥^	P_2r_^♦^	P_2b_^♣^
**ULvW**	0.97	*1.10*	1.10	*1.30 ± 0.10*	1.30 ± 0.09	*1.50*	1.50	1.20	1.30	1.30	1.30
**UBL**	0.91	*17.0*	15.0	*22.0 ± 1.90*	20.0 ± 1.40	*26.0*	23.0	22.0	20.0	23.0	19.0
**UNS**	0.88	*8.70*	9.60	*20.0 ± 3.60*	16.0 ± 2.50	*30.0*	22.0	20.0	17.0	18.0	16.0
**Tacy**	0.88	*16.0*	23.0	*30.0 ± 7.20*	34.0 ± 5.30	*48.0*	46.0	-	32.0	-	35.0
**UBM**	0.77	*1.40*	1.50	*2.40 ± 0.37*	2.0 ± 0.18	*3.30*	2.50	2.20	1.90	2.80	2.0
**UMFM**	0.74	*1.20*	1.40	*2.20 ± 0.36*	1.80 ± 0.18	*3.00*	2.30	2.00	1.80	2.50	1.80
**TY**	0.74	*79.0*	−110	*310 ± 100*	41.0 ± 44.0	*570*	160.0	-	21.0	-	63.0
**UCLP**	0.78	*0.23*	1.00	*1.20 ± 0.36*	1.60 ± 0.23	*1.80*	2.10	0.45	1.40	1.40	1.90
**UCLS**	0.65	*0.40*	0.73	*1.10 ± 0.25*	1.10 ± 0.15	*1.90*	1.60	1.60	1.20	1.20	1.00
**SFY**	0.77	*24.0*	−140	*230 ± 91.0*	34.0 ± 46.0	*460.0*	150	-	8.10	-	60.0
**UKLvW**	0.69	*1.10*	1.20	*1.50 ± 0.09*	1.40 ± 0.05	*1.70*	1.50	1.40	1.40	1.50	1.40
**PFR**	0.73	*9.70*	17.0	*30.0 ± 13.0*	30 ± 7.40	*84.0*	60.0	-	32.0	-	29.0
**UKEC**	0.65	*0.35*	0.60	*0.73 ± 0.06*	0.70 ± 0.03	*0.81*	0.78	0.71	0.71	0.73	0.69
**UNPs**	0.51	*2.80*	2.80	*4.10 ± 0.57*	3.40 ± 0.22	*5.60*	3.90	5.20	3.20	4.40	3.60
**UBW**	0.60	*14.0*	14.0	*17 ± 0.93*	15.0 ± 0.32	*21.0*	16.0	17.0	15.0	18.0	15.0
**UTBM**	0.49	*1.90*	2.40	*4.60 ± 0.96*	3.30 ± 0.32	*7.70*	4.20	4.40	3.0	5.70	3.60
**UKTO**	0.55	*84.0*	85.0	*100 ± 9.40*	92.0 ± 3.50	*130*	100.0	95.0	87.0	100.0	98.0

^*Narrow-sense genomic heritability for trait/model. ^†^Minimum raw trait value. ^‡^Minimum BLUP trait value. ^§^Mean raw trait value ± standard error. ^#^Mean BLUP trait value ± standard error. ⚭Minimum raw trait value. ⚔Minimum BLUP trait value. ♠Maternal raw trait value. ♥Maternal BLUP trait value. ♦Paternal raw trait value. ♣Paternal BLUP trait value^

**Table 3:** Cranberry population CNJ04 trait and BLUP summary for all-year model with heritability above 0.47. Only models with significant (*p* < 0.05) genotypic effects are displayed.

Trait		h^2*^	F_1_ progeny							Parents				
			Min_r_^†^		Min_b_^‡^	μ_r_ ± SE^§^	μ_b_ ± SE^#^	Max_r_^⚭^	Max_b_^⚔^	P_1r_^♠^		P_1b_^♥^	P_2r_^♦^	P_2b_^♣^
**ULvW**		0.96	*1.10*		1.10	*1.2 ± 0.09*	1.2 ± 0.07	*1.5*	1.40	1.40		1.20	-	-
**UBL**		0.90	*19.0*		18.0	*22 ± 1.40*	20 ± 1.10	*27*	24.0	24.0		20.0	-	-
**UNS**		0.88	*8.70*		9.40	*19 ± 3.40*	17 ± 2.40	*26*	22.0	17.0		17.0	-	-
**Tacy**		0.86	*10.0*		25.0	*23 ± 5.80*	34 ± 4.60	*35*	42.0	-		29.0	-	-
**UBM**		0.81	*2.10*		2.00	*2.7 ± 0.27*	2.3 ± 0.17	*3.3*	2.80	2.80		2.30	-	-
**UMFM**		0.83	*1.80*		1.70	*2.4 ± 0.28*	2.0 ± 0.17	*3.3*	2.50	2.30		1.90	-	-
**PAC**		0.73	*1.00*		1.20	*1.4 ± 0.16*	1.4 ± 0.10	*1.8*	1.70	-		1.40	-	-
**TY**		0.62	*63.0*		48.0	*370 ± 89*	180 ± 41.0	*540*	260	-		160	-	-
**UCLP**		0.56	*0.29*		1.10	*1.2 ± 0.35*	1.5 ± 0.14	*1.9*	1.70	1.50		1.40	-	-
**UCLS**		0.65	*0.47*		1.10	*1.3 ± 0.32*	1.5 ± 0.18	*1.9*	1.80	1.30		1.60	-	-
**SFY**		0.50	*58.0*		52.0	*320 ± 82.0*	140 ± 30.0	*500*	210	-		130	-	-
**UKLvW**		0.55	*1.20*		1.20	*1.4 ± 0.07*	1.3 ± 0.03	*1.6*	1.40	1.40		1.30	-	-
**UKEC**		0.51	*0.59*		0.64	*0.72 ± 0.03*	0.68 ± 0.01	*0.79*	0.70	0.72		0.68	-	-
**UNPs**		0.57	*3.10*		3.80	*4.2 ± 0.48*	4.3 ± 0.23	*5.2*	4.70	3.60		4.20	-	-
**UTBM**		0.55	*3.10*		3.90	*5.1 ± 0.81*	4.6 ± 0.38	*7.6*	5.60	4.80		4.30	-	-

^*Narrow-sense genomic heritability for trait/model. ^†^Minimum raw trait value. ^‡^Minimum BLUP trait value. ^§^Mean raw trait value ± standard error. ^#^Mean BLUP trait value ± standard error. ⚭Minimum raw trait value. ⚔Minimum BLUP trait value. ♠Maternal raw trait value. ♥Maternal BLUP trait value. ♦Paternal raw trait value. ♣Paternal BLUP trait value.^

### 3.4 QTL analysis

#### 3.4.1 *CNJ02* population

For the *CNJ02* population, around six QTL per *trait × model* were found for the scanone method and 10 QTL per *trait × model* were found for the stepwiseqtl method. Of the 385 additive scanone QTL found in models with significant genotype effects (*p <* 0.05*)*, 170 were major QTL (*marker PVE ≥ 0.1*). Of the stepwiseqtl additive QTL found in models with significant genotype effects, 150 of 702 were major. The average scanone percent variance explained (*PVE*) per QTL was 9.8%, with an average model *PVE* of 71.5%. The mean stepwiseqtl QTL *PVE* was 7.2%, with an average model *PVE* of 89.9%. A full list of QTL and their effect sizes can be found in **Supplementary Tables S4**, **S5**.

A summary of stable multi-model, within-trait meta-QTL for *CNJ02* are displayed in **Figure 4A**, **Table 4**. The *Tacy* trait displays a prominent meta-QTL on linkage group 3 (position 55.8 cM) with an average paternal effect of −11 mg units per 100 mg fruit. *UBM*, *UTBM*, and *PFR* all have proximal QTL on linkage group 11 (around 30 cM) with *UBM* and *UTBM* having similar average paternal effect sizes of −0.34 g and −0.29 g. A meta-QTL for MFM (per upright) is found very close to the berry mass and upright berry mass on linkage group 11 (position 38.1 cM) with a similar paternal effect size of −0.3 g/fruit. *PFR* has around 10% decrease for both the maternal and paternal effects. A meta-QTL is found very close to the *UBM* and *UTBM* on linkage group 11 (position 38.1 cM), with a similar paternal effect size of −0.3 g/fruit. *SFY* has a meta-QTL on linkage group 12 at 40.4 cM, with a maternal effect size of 10 g/0.09 m^2^ and a paternal effect size of 46 g/0.09 m^2^. *UKLvW* has a modest meta-QTL on linkage groups 3 (position 30.7 cM) and 9 (position 82.7 cM). *UBL* has a meta-QTL on linkage group 10 (position 53.0 cM) with a maternal effect size of −1.8 mm and paternal effect size of 1.1 mm.

**Table 4:** Co-located, stable QTL in cranberry population CNJ02 found in at least three of four BLUP models. Only QTL with $\bar{R^{2}}\geq 10\%$ are shown. Table is arranged in descending order by mean marker variance.

Trait	Models	LG	Position (cM)^*^	R^2†^	-1.5 LOD (cM)^§^	+1.5 LOD (cM)^#^	Methods^‡^	$\bar{AvB}$ ^⚭^	$\bar{CvD}$ ^⚔^	$\bar{Int}$ ^♠^
**Tacy**	2011+ 2012+ 2013+ all years	3	55.8	27.9	52.9	58.6	scanone+ stepwiseqtl	−1.70	−11.0	0.48
**UBM**	2012+ 2013+ all years	11	30.8	20.9	30.3	31.2	stepwiseqtl	−0.06	−0.34	0.06
**PFR**	2011+ 2012+ all years	11	30.8	20.4	30.3	31.2	scanone+ stepwiseqtl	−10.0	−9.90	2.50
**UTBM**	2011+ 2012+ 2013	11	29.8	18.0	28.4	31.2	scanone+ stepwiseqtl	0.08	−0.29	0.05
**UMFM**	2012+ 2013+ all years	11	38.1	17.2	37.5	38.7	scanone+ stepwiseqtl	−0.11	−0.30	0.08
**SFY**	2011+ 2012+ 2013+ all years	12	40.4	16.0	38.1	42.6	scanone+ stepwiseqtl	10.0	46.0	−1.10
**UKLvW**	2011+ 2012+ all years	3	30.7	15.2	28.6	32.8	scanone+ stepwiseqtl	0.04	0.06	0.02
**UKLvW**	2011+ 2013+ all years	9	82.7	13.5	81.2	84.2	scanone	3e-02	0.07	4.2e-03
**UBL**	2012+ 2013+ all years	10	53.0	12.9	51.8	54.2	scanone+ stepwiseqtl	−1.80	1.10	−0.74

^*Mean position of combined QTL. ^†^Mean of variance explained by combined QTL. ^‡^QTL mapping method applied. ^§^1.5 LOD left interval. ^#^1.5 LOD right interval. ⚭Mean maternal effect size: (AC+AD)-(BC+BD). ⚔Mean paternal effect size: (AC+BC)–(AD+BD). ♠Mean interaction effect size: (AC+BD)-(AD+BC).^

#### 3.4.2 *CNJ04* population

Despite having a lower population size (*n =* 67), and thus lower power to find statistically significant QTL, prominent QTL were found in *CNJ04*. An average of three QTL per *trait × model* were found using scanone and nine QTL per *trait × model* were mapped with stepwiseqtl. Of the 90 scanone QTL found in models with significant genotype effects, 69 were major QTL. Of the stepwiseqtl QTL found in models with significant genotype effects, 81 of 365 were major. The scanone average total BLUP variance explained per QTL was 16.9%, with an average BLUP variance explained by all QTL per *trait × model* of 67%. The stepwiseqtl average total BLUP variance explained per QTL was 7%, with an average BLUP variance explained by all QTL per *trait × model* of 96.6%. A full list of QTL and their effect sizes can be found in **Supplementary Tables S6, S7**.

**Table 5**, **Figure 4B** show the inventory of durable multi-model, within-trait meta-QTL for *CNJ04*. *PAC* content has a meta-QTL on linkage group 7 (position 28.7 cM) with a mean maternal effect size of 0.14 mg/g fruit and a mean paternal effect size of 0.19 mg/g fruit. A meta-QTL associated with the trait *UKLvW* can be found on linkage group 11 (position 10.3 cM), with similar maternal and paternal effect sizes of 0.1. A meta-QTL associated with trait *UBL* is located nearby the trait *ULvW* meta-QTL on linkage group 11 (position 16.0cM) and has maternal and paternal effect sizes of 1.1 and 1.3, respectively. A meta-QTL for *UTBM* is found on linkage group 9 (position 82.6cM) with a maternal effect size of 0.44 g fruit/upright and paternal effect size of 0.19 g fruit/upright.

**Table 5:** Co-located, stable QTL in cranberry population CNJ04 found in at least three of four BLUP models. Only QTL with $\bar{R^{2}}\geq 10\%$ are shown. Table is arranged in descending order by mean marker variance. All QTL were reported below were identified with both scanone and stepwiseqtl methods‡.

Trait	Models	LG	Position (cM)^*^	R^2†^	-1.5 LOD (cM)^§^	+1.5 LOD (cM)^#^	$\bar{AvB}$ ^⚭^	$\bar{\text{CvD}}$ ^⚔^	$\bar{Int}$ ^.♠^
**PAC**	2012+ 2014+ all years	7	28.7	26.6	24.3	33.2	0.14	0.19	−0.03
**ULvW**	2011+ 2014+ all years	11	10.3	25.6	9.6	11.1	0.10	0.11	0.020
**UBL**	2011+ 2012+ 2014	11	16.0	20.9	11.7	20.2	1.10	1.30	−2.8e-03
**UTBM**	2011+ 2014+ all years	9	82.6	14.0	80.9	84.2	0.44	−0.19	0.04

^*Mean position of combined QTL. †Mean of variance explained by combined QTL. ‡QTL mapping method applied. §1.5 LOD left interval. #1.5 LOD right interval. ⚭Mean maternal effect size: (AC+AD)-(BC+BD). ⚔Mean paternal effect size: (AC+BC)–(AD+BD). ♠Mean interaction effect size: (AC+BD)-(AD+BC).^

#### 3.4.3 Multi-trait Cross-population meta-QTL

**Figure 5:**
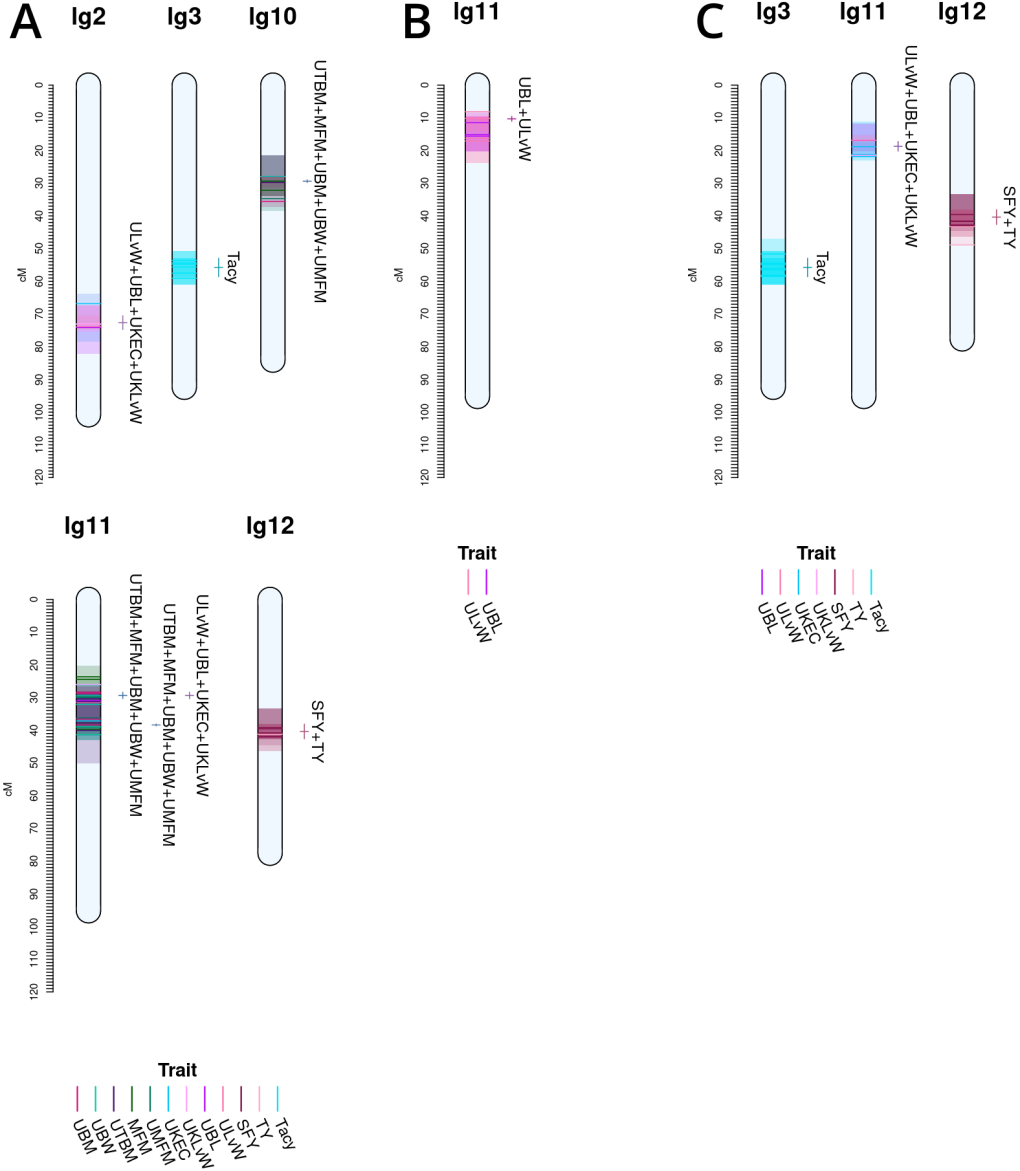
Noteworthy multi-trait, co-located QTL for cranberry populations *CNJ02* **(A)**, *CNJ04* **(B)**, and both *CNJ02* and *CNJ04* **(C)**, based on results from this study.

Co-located trait-marker associations representing stable, cross-population QTL are shown in **Figure 5**. Together, they represent stable meta-QTL derived from the primary QTL found in the current study for *CNJ02*, *CNJ04*, and *CNJ0x*. The traits that pass the filtering constraints outlined in **Table 1A** are all yield or yield-adjacent traits. Composite multi-trait QTL are found on linkage groups 2, 3, 10, 11, and 12 for set *CNJ02* (**Table 1A**, **Figure 5A**); linkage group 11 for set *CNJ04* (**Table 1A**, **Figure 5B**); and linkage groups 3, 11, and 12 for set *CNJ0x* (**Table 1A**, **Figure 5C**).

**Figure 6:**
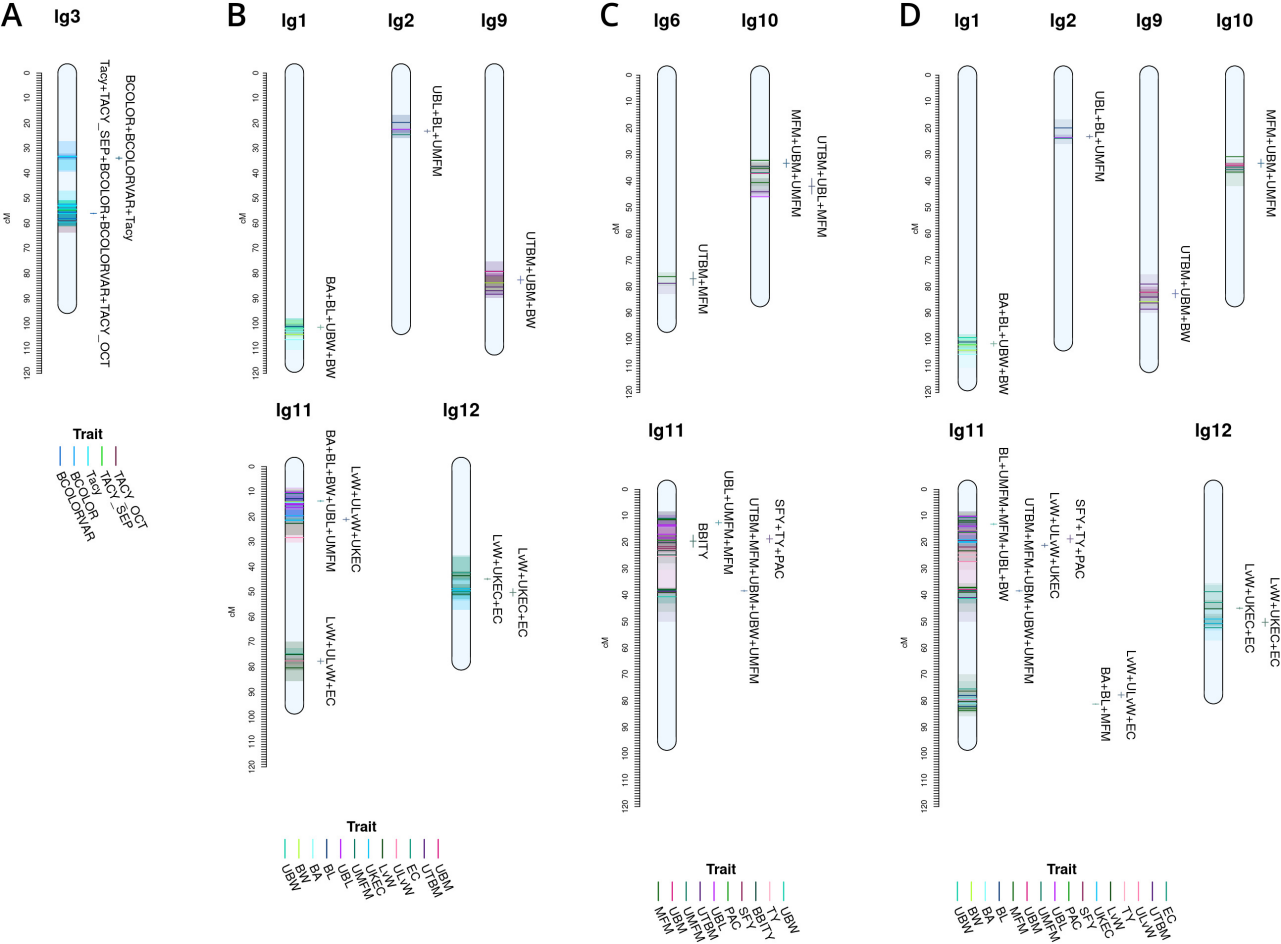
Cross study co-located meta-QTL found comparing QTL from this study with QTL from Diaz-Garcia et al. (2018b) **(A)**, Diaz-Garcia et al. (2018a) **(B)**, Schlautman et al. (2015) **(C)**, and a combined synthesis of Diaz-Garcia et al. (2018a) and Schlautman et al. (2015) **(D)** in cranberry populations *CNJ02*, *CNJ04*, and *GRYG*.

**Figure 7:**
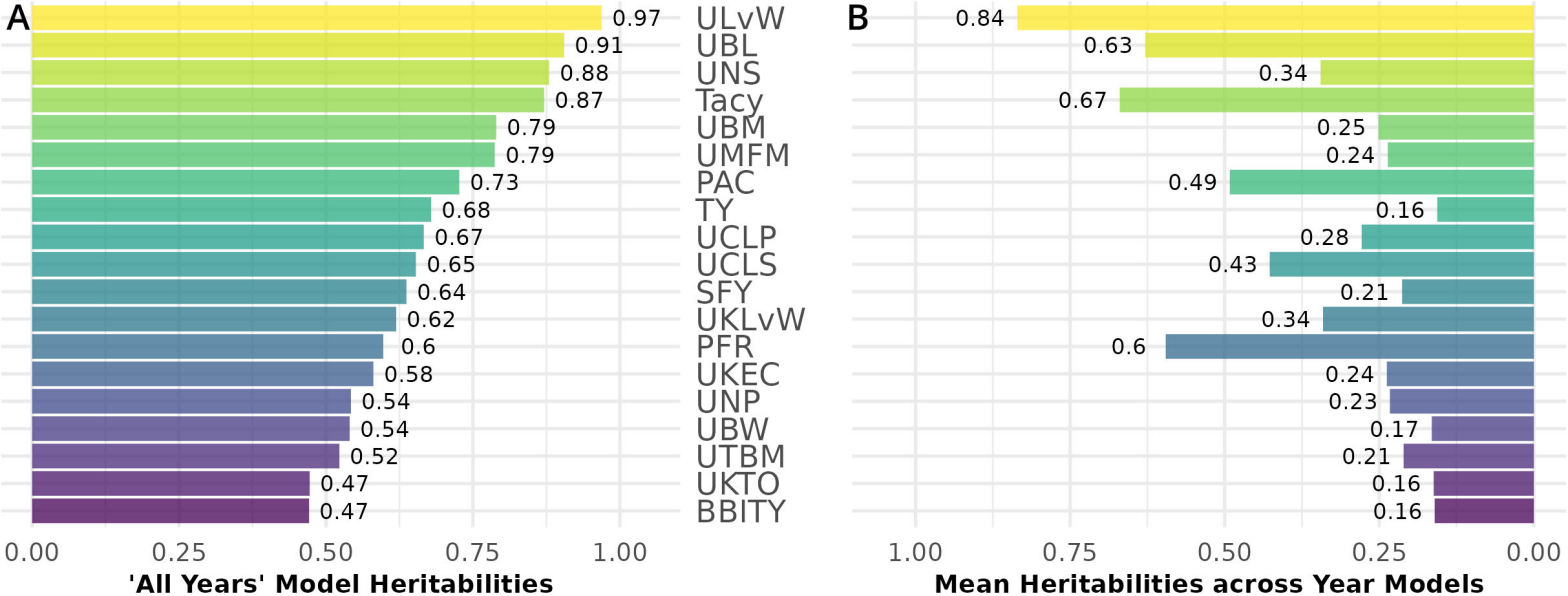
Mean heritability between cranberry populations *CNJ02* and CNJ04 of all-year model **(A)** and across within-year models (**B**). Only the top 50% of traits based on mean within-year model heritability are displayed.

**Figure 8:**
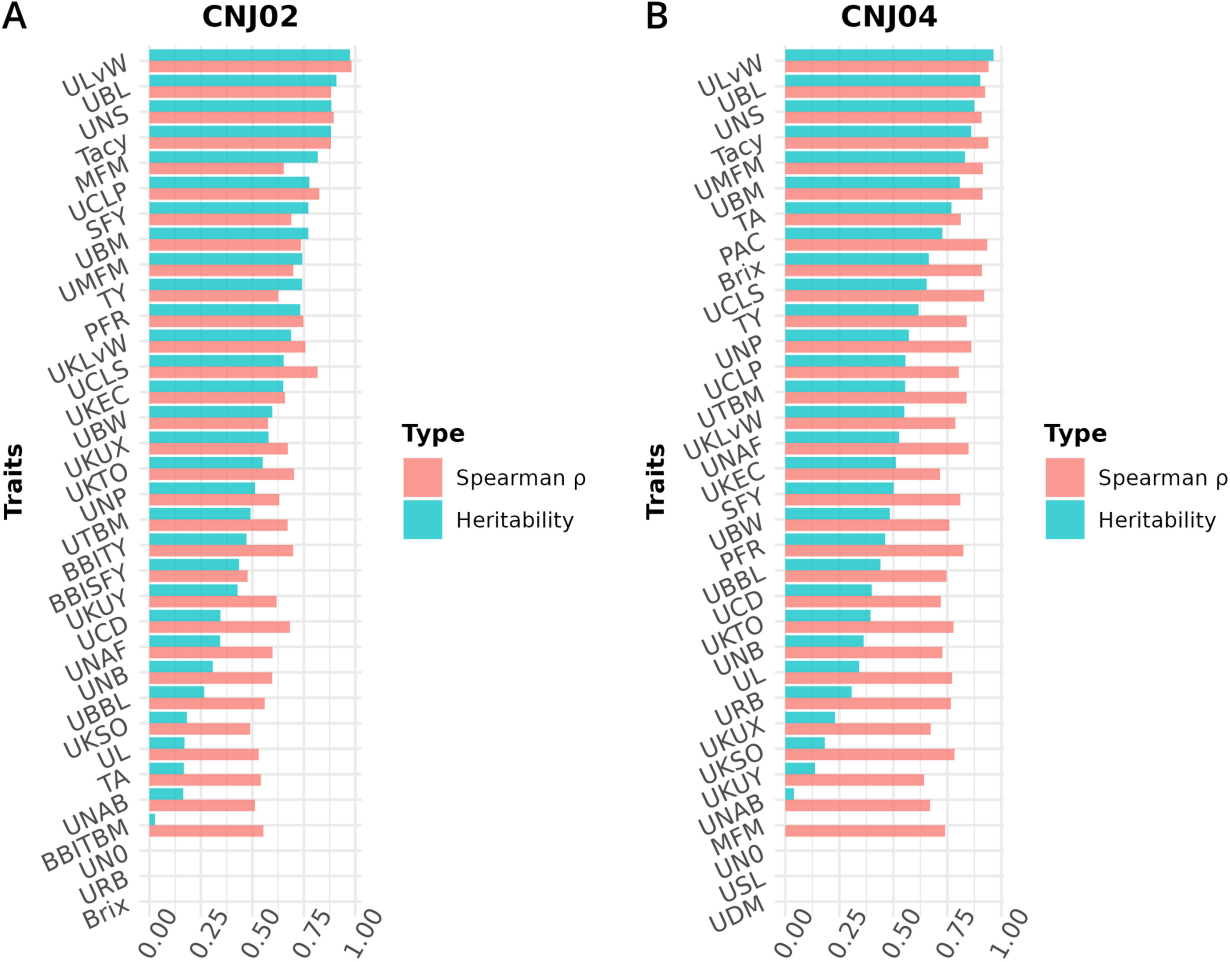
Heritabilities and Spearman’s rank correlation coefficients ( ρ ) for cranberry traits in populations **(A)** *CNJ02* and **(B)** *CNJ04*. Correlation coefficients represent the associations between genotype BLUPs and their associated phenotypes for the all-year mixed model.

For set *CNJ02* (**Table 1A**, **Figure 5A**), linkage group 2 has a composite QTL for {*UBL*, *ULvW*, *UKLvW*, *UKEC*} (position 72.6 cM); linkage group 3 has a year-stable meta-QTL for *Tacy (*position 55.8 cM)*;* linkage group 10 has a compound QTL for {*UBM*, *UTBM*, *UBW*, *MFM* and *UMFM*} (position 29.4 cM); linkage group 11 has three meta-QTL at two positions for {*MFM*, *UMFM*, *UTBM*, *MFM*, *BW*} and {*UBL, ULvW, UKEC, UKLvW*} (position 29.4 cM) and {*UBM*, *UTBM*, *UMFM*, *MFM*, *UBW*} (position 38.4cM); and, last, linkage group 12 has a co-located QTL for {*SFY*, *TY*} (position 40.4 cM). Set *CNJ04* (**Table 1A**, **Figure 5B**), linkage group 11 has a co-located QTL for {*UBL, ULvW*} (position 10.3 cM). For set *CNJ0x* (**Table 1A**, **Figure 5C**), linkage group 3 has a meta-QTL for *Tacy* (position 55.8 cM); linkage 11 has compound QTL for upright traits {*UBL*, *ULvW*, *UKEC*, *UKLvW*} (position 18.7 cM); and linkage 12 has a composite QTL for {*SFY*, *TY*} (position 40.4 cM).

**Figure 6** shows meta-QTL found through comparison of this study’s QTL to QTL previously found in Diaz-Garcia et al. (2018a); Diaz-Garcia et al. (2018b), and Schlautman et al. (2015), collectively containing meta-QTL from populations *CNJ02*, *CNJ04*, and the additional linkage mapping population *GRYG*. Cross-study meta-QTL co-located with populations *CNJ02* or *CNJ04* include those associated with sets *CNJ0x &* Diaz-Garcia, 2018b (**Table 1B**; **Figure 6A**); *CNJ0x &* Diaz-Garcia, 2018a (**Table 1B**; **Figure 6B**); *CNJ0x &* Schlautman 2015 (**Table 1B**; **Figure 6C**), and *CNJ0x &* Schlautman, 2015 *&* Diaz-Garcia, 2018a (**Table 1B**; **Figure 6D**; **Supplementary Table S2**). The shared set *CNJ0x &* Diaz-Garcia, 2018b (**Figure 6A**) contains meta-QTL associated with chemistry traits, yield traits, and color traits derived from digital images of berries. Color traits are tightly associated with *Tacy* content (Diaz-Garcia, 2018b). Set *CNJ0x &* Diaz-Garcia, 2018b has two composite QTL on linkage group 3 that are stable across this study and the Diaz-Garcia et al. (2018b) QTL study for composite traits {*BCOLOR, Tacy, BCOLORVAR}* (34.0 cM) and {*Tacy, BCOLOR, TACY_SEP, BCOLORVAR, TACY_OCT*} (56.1 cM). Set *CNJ0x &* Diaz-Garcia, 2018a (**Figure 6B**) contains meta-QTL for traits affiliated with yield and berry quality, including digital traits measuring berry shape and size parameters (Diaz-Garcia et al., 2018a). For set *CNJ0x &* Diaz-Garcia, 2018a, linkage group 1 has a composite QTL for {*BA*, *BL*, *UBW*, *BW*} (102 cM); linkage group 2 has a meta-QTL for {*upright berry length* (*UBL*), *BL, UMFM*} (position 23.3 cM); linkage group 9 has a composite QTL for {*UTBM*, *UBM*, *BW*} (82.7 cM); linkage group 11 has three meta-QTL for {*BA*, *BL*, *BW, UBL, UMFM*}, {*LvW*, *ULvW*, *UKEC*}, and {*LvW*, *ULvW*, *EC*} clustered at positions 13.8, 21.1, and 77.8 cM, respectively; and linkage group 12 has two separate but close clusters of meta-QTL for {*LvW*, *UKEC*, *EC*} at positions 44.9 and 50.3cM, respectively. Set C*NJ0x & Schlautman* (**Figure 6C**), linkage group 6 has a compound QTL for {*UTBM*, *MFM*} (position ~77.0 cM*)*; linkage group 10 has two meta-QTL for {*UBM, MFM, UMFM*} (positions 33.3 and 42.0 cM), linkage group 11 has four composite QTL for {*UBL, MFM, UMFM)}* (12.6 cM), {*PACs*, *SFY*, *TY*} (18.7 cM), multi-year {*BBITY*} (19.6 cM), and for {*UBM, UTBM*, *MFM*, *UMFM*, *UBW*} (38.4 cM). For set C*NJ0x &* Schlautman, 2015 *&* Diaz-Garcia, 2018a (**Figure 6D**), linkage group 1 has a compound QTL for {*berry area* (*BA*), *BW*, *UBW*, *BL*} (position 101.6 cM); linkage group 2 has a meta-QTL for {*BL*, *UBL*, *UMFM*} (position 23.2 cM); linkage groups 9 and 10 each have a single meta-QTL—linkage group 9 for {*UBM*, *UTBM*, *BW*} (position 82.7 cM) and linkage group 10 for {*UBM*, *MFM*, *UMFM*} (position 33.3 cM); linkage group 11 has the highest number and density of meta-QTL—a cluster of three composite QTL (13–21 cM), one meta-QTL (38.4 cM), and two co-located QTL (77.8 cM to 81.2 cM). The first cluster has meta-QTL at 13.1 cM and 16.0 cM for {*UBL*, *MFM*, *UMFM*, *BW*, *BL*} and {*EC*, *ULvW*} respectively, and meta-QTL at 18.7 cM and 21.1 cM for {*PAC*, *SFY*, *TY*} and {*UKEC*, *LvW*, *ULvW*}. Linkage group 11 also has compound QTL for {*UBM*, *UTBM*, *MFM*, *UMFM*, *UBW*} (38.4 cM), {*EC*, *LvW*, *ULvW*} (77.8 cM), and {*BA*, *BL*, *MFM*} (81.2 cM); and linkage group 12 has two meta-QTL for {*LvW*, *EC*, *UKEC*}—one at 45.0cM and one at 50.3 cM.

## 4 Discussion

A recent acceleration of the quantity and quality of cranberry molecular resources has propelled advances in cranberry breeding (Vorsa and Zalapa, 2019; Diaz-Garcia et al., 2020). Advances in the genetic capital of this important fruit crop include development of high-density linkage maps, construction of mitochondrial, and nuclear genome assemblies, a plethora of QTL mapping studies in a variety of important traits (Vorsa and Zalapa, 2019), and a feasibility study in genomic prediction and genomic selection (Covarrubias-Pazaran et al., 2018).

Shifts and innovations in cranberry phenotyping methods have paralleled advances in genetic resource development. Up until 60 years ago, breeders would select cranberry breeding material based on traits measured from the fundamental unit of cranberry productivity: the “reproductive upright.” Since then, modernization of farming technology and management methods, improved understanding of cranberry physiology, emerging cranberry products and markets, expansion of high-yielding cultivars, and climate change have transformed how breeders prioritize and assess traits (Vorsa and Zalapa, 2019). The focus has thus shifted from assessing cranberry uprights to assessing plot-level measurements, increasingly combined with high-throughput digital imaging (Diaz-Garcia et al., 2018a, 2018b).

Despite a widespread acceptance and application of these modern trait collection methods in cranberry breeding, a translation gap exists in how upright traits relate to these newer phenotype scoring methods. With dense marker maps and parallel collection of both reproductive upright attributes and plot-level traits, the current study offers a unique opportunity to use both phenotypic paradigms for genetic mapping. This paper is the first to comprehensively report correlations, heritabilities, and QTL based on both traditional and modern phenotyping methods.

### 4.1 Trait correlations

In the *CNJ02* population, *Tacy* and *PAC* are negatively correlated with *SFY* but positively correlated with *PFR*. These correlations indicate that, as pigments and other flavonoids develop, cranberry fruit develop more decay, which is likely a consequence of biochemical patterns that coordinate with the timing of cranberry ripening, over-ripening, and subsequent rot (**Supplementary Table S8**) (Georgi et al., 2013; Daverdin et al., 2017). We also found that, in both studied populations, *MFM*—a plot yield trait—was strongly correlated with many of the per upright yield-related traits (e.g., *UBL*, *UBM*, and *UBW*). These correlations are the first reported link between a plot-level yield trait and per-upright yield traits in both *CNJ02* (**Figures 3A, B**; **Supplementary Table S8**) and *CNJ04* (**Figures 3C, D**; **Supplementary Table S9**). In the *CNJ02* population, modest but significant positive correlations between plot trait *TY* and upright traits {*UTBM, UBW, UBM, UMFM, UNP*s, and *UNBs*} further links plot traits with upright traits. These plot yield × upright trait correlations (*TY* × {*UNPs*, *UNB*, *UTBM*}) were also observed in the *CNJ04* population, though to a lesser degree (**Figures 3C, D**; **Supplementary Table S9**). These correlations not only establish important relationships between plot level and upright trait types but also may indicate the existence of co-located trait blocks within the genome (Diaz-Garcia et al., 2018a, 2018b). For both the *CNJ02* and *CNJ04* populations, we found that *UKLvW*, a trait derived from berry shape categorical data, had a moderate positive correlation with quantitative traits such *ULvW*. This result validates that berry chimeras composited from the berry shape scores track well with numerically precise, accurately measured traits such as *ULvW*, despite being a subjective trait prone to imprecision.

### 4.2 Heritabilities

Estimating heritabilities offers breeders a chance to find confidence that their selections will be fruitful in subsequent generations. Although this study calculates heritabilities from mixed model estimates of environmental, additive genetic, and residual variances, the heritabilities derived offer breeding insights when selecting upon model derived BLUPs. Many of the calculated heritabilities in this study are comparable to the results of other studies. For example, the high mean heritability of the *ULvW* (*ULvW ratio*, $h^{2}\approx 0.97$ ) parallels plot data findings found in *GRYG*, a population genetically distinct from *CNJ02* and *CNJ04* (Diaz-Garcia et al., 2018a). Consistently high heritability across these three populations suggests a strong genetic persistence of *ULvW ratio*. High heritabilities in both *ULvW ratio* and in related plot traits indicates that selecting rounder berries (low *ULvW ratio)* with either method is effective. Moderate heritability estimates of *TY* ( $h^{2}\approx 0.74$ for *CNJ02*, $h^{2}\approx 0.62$ for *CNJ04*) are lower relative to other berry size and weight parameters – these estimates are consistent with the highly polygenic nature of this trait in other crops. Schlautman et al. (2015) estimated *CNJ02* heritabilities of $h^{2}\approx 0.70$ and $h^{2}\approx 0.64$ for mean fruit weight (*MFM*) and *TY*, respectively. Trait heritabilities for *Tacy*, *Brix*, and *titratable acidity (TA)* were consistent with the present study, while their lower heritability estimates for *TY* ( $0.29<\;h^{2}<0.47$ ) are likely due to the use of small populations (Vorsa and Johnson-Cicalese, 2012). Furthermore, a study by Johnson-Cicalese et al. (2015) using midparent-progeny mean regression estimates of heritability for fruit rot resistance ( $h^{2}\approx 0.81$ ) found consistently higher heritability with *fruit rot (%) (PFR)* in the *CNJ02* population of this study ( $h^{2}\approx 0.73$ ). Lower heritability estimates found in *CNJ04* ( $h^{2}\approx 0.46$ ) could be due to reduced statistical power from lower population size. Moderate heritability of traits like *SFY* and *PFR* demonstrate limited but possible potential for selecting rot-resistant varieties in regions where berry rot is a problem. The traits *TA* and *Brix* exhibited notable differences in heritability between *CNJ02* and *CNJ04*. In *CNJ04*, the heritabilities of *TA* ( $h^{2}\approx 0.77$ ) and *Brix* ( $h^{2}\approx 0.66$ ) were rather high, while in *CNJ02*, these two traits exhibited low heritability (*TA*, $h^{2}\approx 0.17$ and *Brix*, $h^{2}\approx 0$ ). This likely has to do with differences in the standing genetic variation between the parents for each population. For *CNJ02*, the parents (cv. *Mullica Queen^®^* and cv. *Crimson Queen^®^*) are highly elite third-generation hybrids with little distinct genetic variation for *Brix* and *TA*, thus exhibiting low heritability in their progeny. In contrast, *CNJ04* has one highly elite parent (cv. *Mullica Queen^®^*), while the other parent (cv. *Stevens*) is a first-generation hybrid. Since *CNJ04’s* two parents are genetically and phenotypically distinct and consequently have more standing genetic variation between the parents, these traits manifest higher heritability for *Brix* and *TA*.

The heritabilities presented here may have been affected by population sizes, reduced recombination history in F_1_ mapping populations, and higher degrees of freedom when working with heterozygous, four-way crosses, and there is lower statistical power to segregate genetic from phenotypic variances in mixed models in traits such as *TY*. However, the heritability estimates were overall consistent in rank relative to other traits measured in recent cranberry studies.

### 4.3 QTL summary

This analysis is one of the most comprehensive QTL mapping studies in cranberry, given the number of traits assessed, the number of factors modeled, and cross-study comparisons. **Figure 5** highlights salient, multi-trait co-located QTL in population sets *CNJ02*, *CNJ04*, and *CNJ0x* (panels **A**, **B**, and **C**, respectively) of the current study. Multi-trait clusters among non-synonymous traits likely represent tight linkage or pleiotropy. Multiple linkage associations found on chromosomes 2, 3, 10, 11, and 12 together constitute traits important to both berry quality and yield. A lower fruit length versus width ratio and a higher MFM translates to larger, spherical berries—quality traits important in SDC production. Quality traits relevant to SDC production—*ULvW*, *UKLvW*, *UKEC*, and *UMFM* and *MFM*)—have stable and co-located QTL on linkage groups 2, 11, and 12. *CNJ02* also displays a modest composite QTL {*SFY*, *TY*} on linkage group 12, where *SFY* is an important measure of rot. Two co-located meta-QTL on linkage group 11 ({*UBM* and *UTBM*)*, MFM, UMFM, UBW*} and {*UBL, ULvW, UKLvW, UKEC*} are likely identical QTL or the result of pleiotropy (position 29.4 cM). *CNJ04* only displays one robust meta-QTL on linkage group 11 for {*UBL*, *ULvW*} at position 10.32 cM. Despite a lack of a multi-year stable QTL for *Tacy* in *CNJ04*, the stable meta-QTL on linkage group 3@55.8 cM in *CNJ02* is also shared with a *CNJ04* QTL found in the *all-year* model. This shared cross-population *Tacy* QTL on linkage group 3 indicates the importance of this region to *Tacy* production, likely from the shared parent *Crimson Queen^®^* common to both *CNJ02* and *CNJ04*. The meta-QTL {*UBL*, *ULvW*, *UKEC*, *UKLvW*} common to both *CNJ02* and *CNJ04* (**Figure 5C**) on linkage group 11@18.7 cM lacks the position stability relative to the same multi-trait meta-QTL found in population *CNJ02* on linkage group 11@29.4 cM (**Figure 5A**). This position shift from 11@29.4 cM to 11@18.7 cM is consistent with the larger LOD 1.5 interval for the elemental trait QTL, but the relative cross-population stability still highlights the importance of this region to the composite {*UBL*, *ULvW*, *UKEC*, *UKLvW*}, with most of the region’s genetic variation likely driven by *UBL*. As with Tacy on linkage group 3, a meta-QTL for {*SFY*, *TY*} on linkage group 12@40.4cM is found both in *CNJ02* (**Figure 5A**) alone and in the combined analysis of *CNJ02* and *CNJ04* (**Figure 5C**), indicating the cross-population stability of this QTL.

**Figure 6A** features prominent, multi-study, multi-year, and multi-trait QTL from the set *CNJ0x &* Diaz-Garcia et al., 2018b. Tacy and other color-relevant meta-QTL on linkage group 3@56.1cM in populations *CNJ02* and *CNJ04* is consistent with the results found in **Figure 5C**. This meta-QTL also demonstrates consistency across two separate studies, underpinning the importance of this genomic region to *Tacy* and color development in *CNJ02* and *CNJ04*. Evidence of an additional meta-QTL on linkage group 3@34.0 cM in population *GRYG* and *CNJ04* indicates an alternative genomic region responsible for regulating fruit color in a distinct population set. This is consistent with previously observed genomic regions that encompass QTL for many related fruit quality traits in cranberry for fruit quality traits

Comparing the QTL found in the current study against Diaz-Garcia et al. (2018a) offers a unique perspective to compare berry size parameter QTL found using newer digital imaging techniques against parameters assayed using the manually measured traits pertinent to this study (**Figure 6B**). The composite meta-QTL on linkage group 1@101.7cM establishes a correspondence of *UBW* (current study) with *BW* and *berry length* (*BL*) (*digital traits;* Diaz-Garcia et al., 2018a). *UBL* (current study) coincides with *BL* (digital trait; Diaz-Garcia et al., 2018a) at meta-QTL found in linkage groups 2@23.2 cM and 11@13.8 cM for populations *CNJ04* and *GRYG*. Meta-QTL associated with round, spherical berries that also demonstrate coincidence between manual and digital traits are found on linkage groups 11 and 12. A meta-QTL in linkage group 11@21.1cM connects the current study traits *UKEC* and *ULvW* with the image-derived digital trait *berry length:width ratio* (LvW; Diaz-Garcia et al., 2018a). The composite QTL at 11@77.8cM links the digital traits *LvW* and *berry shape eccentricity* (*EC*; Diaz-Garcia et al., 2018a) to *ULvW* (current study) across populations *GRYG* and *CNJ02*. Two meta-QTL for composite trait {*LvW*, *UKEC*, *EC*} on linkage group 12, positions 45.0 cM and 50.3 cM, relate upright trait (*UKEC*) with digitally measured traits {*LvW*, *EC*} (Diaz-Garcia et al., 2018a) across populations *GRYG* and *CNJ04*. These QTL complexes on linkage groups 11 and 12 together highlight the interchangeability of two distinct phenotyping (upright vs. plot) methods across distinct populations.

QTL reported by Schlautman et al. (2015) are associated with plot yield traits and meta-QTL shown in **Figure 6C** demonstrate the coincidence of these QTL with QTL found in the current study. The meta-QTL on linkage group 6@77.0cM ties UTBM (current study) to *MFM* (Schlautman et al., 2015), consistent with the high correlation (*p =* 0.71) between these two traits (**Supplementary Table S8**). Linkage group 10 has two meta-QTL that demonstrate a correspondence of *MFM* (Schlautman et al., 2015) with {*UBM*, *UMFM*} (current study; position 33.3 cM) and {*UTBM*, *UBL*} (current study; position 42.0). Linkage group 11 contains several meta-QTL that associate the yield related plot traits *biennial-bearing index - total yield* (*BBITY*), *TY*, and *MFM* (Schlautman et al., 2015) with upright traits *UBL*, *UMFM*, *UTBM*, *UBM*, and *UBW*, further establishing the congruity between plot traits and upright traits.

A comprehensive comparison of cross-study meta-QTL is shown in **Figure 4D**. The meta-QTL displayed include compound, stable QTL found across up to three independent studies (current study; Schlautman et al., 2015; Diaz-Garcia et al., 2018a). QTL found in **Figure 5D** are duplicated in **Figure 5B**, apart from a three-study meta-QTL on linkage group 11@13.1 cM for the compound trait {*UBL*, *MFM*, *UMFM*, *BW*, *berry length* (*BL*)}, which differs inappreciably from the meta-QTL found for composite trait {*berry area* (*BA*), *UBL*, *UMFM*, *BW*, *BL*} on linkage group 11@13.8cM (**Figure 4B**). This meta-QTL for linkage group 11, position 13.1cM includes a *MFM* QTL discovered using a different dataset and different methodology by Schlautman et al. (2015). Consequently, this genomic region shows evidence of a multi-study, multi-trait, multi-year QTL for mean berry size. Other multi-study meta-QTL that include QTL from Schlautman et al. (2015) include upright traits {*UBM*, *UMFM*} and plot trait *MFM* on 10@33.3cM, additional evidence of an association of *PAC* with *SFY* and *TY* on 11@18.7cm, upright traits {*UBM*, *UTBM*, *UMFM*, *UBW*} (current study) with plot trait *MFM* (Schlautman et al., 2015) on 11@38.4cM, and digital traits {*BA*, *BL*} (Diaz-Garcia et al., 2018a) with plot trait *MFM* (Schlautman et al., 2015) on 11@81.2cM.

### 4.4 Conclusion

Traditionally, marker-trait associations derived from QTL or GWAS studies are validated using advanced molecular techniques such as genetic engineering approaches to compare knockout and knockdown mutant phenotypes. However, without efficient transformation methods, lack of protocols to re-differentiate callus tissue in culture, and an absence of research on genetically malleable genotypes, these traditional marker-trait validation methods are currently impossible to implement in cranberry. As such, the results of additional cranberry trait-mapping studies in new environments (locations and years), and their comparison to other studies’ discoveries, can serve as a viable method to fortify consistent findings and validate distinct phenotyping methods measuring analogous traits. We report here coherent results that have emerged across multiple studies to provide important targets for marker assisted selection (MAS).

This study set out to characterize and assess for the first time the genetic basis of numerous cranberry reproductive upright traits in conjunction with many plot-level and other modern phenotyping traits. The existence of strong correlations between reproductive upright traits (classical) for berry parameters and plot-level traits (modern) for yield demonstrate that more efficient, modern phenotyping methods can act as relevant proxies in QTL mapping studies. Roughly comparable heritabilities between analogous traits and consistent meta-QTL found in advanced phenotyping studies such as Diaz-Garcia et al. (2018b; 2018a), **Figures 6**–**8**, further demonstrate the near parity of older scoring techniques vis-à-vis modernized phenotyping methods.

As the time needed to measure and score classical upright traits is monumental, requiring hundreds of person hours, the use of combinations of relevant classical and modern phenotyping methods can serve to save time and increase throughput. Moreover, although some of the upright traits collected may provide a unique picture of the genetics and physiology of complex traits, they can still fail to capture certain dimensions of traits, such as TY. This is evident in how a modestly positive correlation between yield and berry size parameters loses most of its association when mapping the genetic basis of these traits. Altogether, classical upright traits when used along with plot-sampled and other modern traits (e.g., digital imaging processing) provide a more detailed picture of genotypic performance for traits important to growers, breeders, and the application of genetic studies such as QTL mapping.

## Data Availability

All raw data can be found on the Genome Database for Vaccinium at https://www.vaccinium.org/bio_data/7547154. Software to generate BLUPs, QTL, and meta-QTL are available at https://github.com/bliptrip/CNJ0x-Trait-Mapping.
