# Supplementary material for: Of buds and bits: a meta-QTL study identifies stable QTL for berry quality and yield traits in cranberry mapping populations (Vaccinium macrocarpon Ait.)
Source: Front Plant Sci. 2024 Sep 17;15:1294570. doi: 10.3389/fpls.2024.1294570 (PMC11442229; doi:10.3389/fpls.2024.1294570)
Supplement: Supplementary file 7 [file Table1.docx]

***Supplementary Material***

**Of buds and bits: a meta-QTL study identifies stable QTL for berry quality and yield traits in cranberry mapping populations (*Vaccinium macrocarpon* Ait.)**

Andrew F. Maule^1,2*^, Jenyne Loarca^1,2^, Luis Diaz-Garcia^3^, Hector Lopez-Moreno^1,2^, Jennifer Johnson-Cicalese^4,5^, Nicholi Vorsa^4,5^, Massimo Iorizzo^6,7^, Jeffrey L. Neyhart^8^, Juan Zalapa^2*^

^1^ Department of Plant and Agroecosystem Sciences, University of Wisconsin-Madison, Madison WI, United States

^2^ USDA-ARS, Vegetable Crops Research Unit, Madison WI, United States

^3^ Department of Viticulture and Enology, University of California-Davis, One Shields Avenue, Davis CA, United States
^4^ P.E. Marucci Center for Blueberry and Cranberry Research and Extension Center, Rutgers University, Chatsworth NJ, United States
^5^ Department of Plant Biology, School of Environmental and Biological Sciences, Rutgers University, New Brunswick NJ, United States
^6^ Department of Horticultural Science, North Carolina State University, Raleigh NC, United States
^7^ Plants for Human Health Institute, North Carolina State University, Raleigh NC, United States
^8^ United States Department of Agriculture-Agricultural Research Service, Genetic Improvement for Fruits & Vegetables Laboratory, Chatsworth NJ, United States

**^*^ Correspondence:**Juan Zalapa ([jezalapa@wisc.edu](mailto:jezalapa@wisc.edu), [juan.zalapa@usda.gov](mailto:juan.zalapa@usda.gov)); Andrew Maule ([maule2@wisc.edu](mailto:maule2@wisc.edu))

# Supplementary Figures and Tables

## Supplementary Figures

## Supplementary Tables

Supplementary Table S1. Summary of traditional (upright and berry) and plot traits collected from cranberry (*Vaccinium macrocarpon* Ait*.*) populations *CNJ02* and *CNJ04* in years 2011-2014.

| **Trait^a^** | **Abbv^b^** | **Description** |
| --- | --- | --- |
| **Berry Shape Chimera Parameters** |  |  |
| UMCC - X-axis^*^ | *UKUX*^*^ | log of Unsigned Manhattan Chain Code - X-axis. Larger values indicate more change in berry curvature. |
| UMCC - Y-axis^*^ | *UKUY*^*^ | log of Unsigned Manhattan Chain Code - Y-axis. Larger values indicate more change in berry curvature. |
| Eccentricity^*^ | *UKEC*^*^ | Mathematical eccentricity of derived berry chimera shape. Circular berries have 0 eccentricity, elliptical berries have larger eccentricities the more oblong the berry is, but less than 1. |
| Length:Width Ratio^*^ | *UKLvW*^*^ | The derived berry chimera shape length versus width ratio. |
| Tortuosity^*^ | *UKTO*^*^ | The derived berry chimera shape tortuosity. Larger values indicate increased ‘waviness’ in the berry contour. Smoother/rounder berries will have lower values. |
| Solidity^*^ | *UKSO*^*^ | The derived berry chimera shape density relative to its convex hull. Lower values indicate more ‘waviness’ in the berry contour, while higher values have smoother contour curvature. |
| **Largest Berry Traits** |  |  |
| Length ($mm$) | *UBL* |  |
| Width ($mm$) | *UBW* |  |
| Mass ($g$) | *UBM* |  |
| Calyx Diameter ($mm$) | *UCD* |  |
| Calyx Lobe Fold Pattern ($open;medium;closed$) | *UCLP* |  |
| Calyx Lobe Size ($small;medium;large$) | *UCLS* |  |
| Calyx End Shape ($round;flattened;pointed$) | *UCES* |  |
| Berry Pedicel End Shape ($round;flattened;pointed$) | *UBES* |  |
| Berry Skin Bloom Level ($shiny;moderate;heavy$) | *UBBL* | Assesses the amount of wax on the berry surface. |
| Berry Shape ($round;oval;oblong;$  $pyriform;spindle$) | *UBS* |  |
| Number Developed Seeds | *UNS* |  |
| Length:Width Ratio^*^ | *ULvW*^*^ | Derived from UBL / UBW |
| **Plot Traits** |  |  |
| Total Yield ($\frac{g}{{ft}^{2}}$) | *TY* |  |
| Sound Fruit Yield ($g$) | *SFY* |  |
| Mean Fruit Mass ($\frac{g}{berry}$) | *MFM* | Calculated based on random sample of 100 berries. |
| Percent Fruit Rot ($\frac{100*\left( TY-SFY \right)}{TY}\%$**)** | *PFR* |  |
| Total Anthocyanins ($\frac{mg}{100g berries}$) | *Tacy* |  |
| °Brix (*Soluble Solids* $\%$) | *Brix* |  |
| Titratable Acidity ($\%$) | *TA* |  |
| Proanthocyanins ($\frac{mg}{g berries}$) | *PAC* |  |
| **Upright Traits** |  |  |
| Total Berry Mass ($g$) | *UTBM* |  |
| Upright Length ($cm$) | *UL* |  |
| Upright Secondary Length | *USL* | Secondary vegetative growth on upright. |
| Dry Mass of Leaves ($g$) | *UDM* |  |
| Rebud ($yes;no$) | *URB* | Evidence for rebud behavior, where a fruiting upright has a larger bud than a vegetative upright. |
| Number of Pedicels | *UNP* |  |
| Number Pedicels without Fruit | *UN0* |  |
| Number Pedicels with Mature Berries | *UNB* |  |
| Number Pedicels with Aborted Flowers | *UNAF* |  |
| Number Pedicels with Aborted Berries | *UNAB* |  |
| Upright Mean Fruit Mass^*^ ($\frac{g}{berry}$) | *UMFM*^*^ | Derived from UTBM / UNB |
| **Biennial Bearing Traits^†^** |  |  |
| Biennial Bearing Index – Upright Total Berry Mass^*^ | *BBIUTBM*^*^ | $BBIUTBM = \left\vert100 \times\frac{\Delta UTBM_{successive}}{\sum UTBM_{successive}} \right\vert$ |
| Bienniel Bearing Index – Total Yield^*^ | *BBITY*^*^ | $BBITY = \left\vert100 \times\frac{\Delta TY_{successive}}{\sum TY_{successive}} \right\vert$ |
| Bienniel Bearing Index – Sound Fruit Yield^*^ | *BBISFY*^*^ | $BBISFY = \left\vert100 \times\frac{\Delta SFY_{successive}}{\sum SFY_{successive}} \right\vert$ |
| ^a^Upright Traits were measured on 10 fruiting-uprights. Largest berry traits represent phenotypic values of the largest berry on a given fruiting upright. Plot traits were collected by harvesting berries from a 0.09 m^2^ plot sample and running appropriate assays onsite at Marucci Center. Upright Traits and Largest Berry Traits were collected at Rutgers and assayed at UW-Madison. Trait abbreviations are used to label subsequent figures. Berry Shape Chimera Parameters were derived from canonical representations of berry shapes. Expressions between parentheses indicate trait units or categorical values. | | |
| ^b^Trait abbreviations are used to label subsequent figures. | | |
| ^*^Trait is derived, or calculated, from other measured traits. | | |
| **^†^**Measure of yield stability over successive years, as described in Schlautman *et al.* (2015). | | |

Supplementary Table S2. Abbreviations and descriptions for relevant cranberry (*Vaccinium macrocarpon* Ait*.*) digital and plot traits referenced from Diaz-Garcia *et al.* (2018a), Diaz-Garcia *et al.* (2018b), and Schlautman *et al.* (2015).

| **Trait^a^** | **Abbv** | **Description** |
| --- | --- | --- |
| ***Diaz-Garcia et al. (2018a)*** |  |  |
| Berry Area | *BA* | Berry Area |
| Berry Length | *BL* | Berry length digital measure (major axis diameter of berry contour) |
| Berry Width | *BW* | Berry width digital measure (minor axis diameter of berry contour) |
| Eccentricity | *EC* | A value of 0 represents a perfect circle and a value of 1 represents an ellipse stretched infinitely long (straight line). |
| Berry Length vs. Width | *LvW* | Ratio of berry length to width (major/minor axis lengths). |
| Persistent Homology -  Principal Component 1 | *PH_PC1* | Moderate-to-high correlation with LvW and EC. |
| Persistent Homology -  Principal Component 2 | *PH_PC2* | Moderate-to-low correlation with LvW and EC. |
| ***Diaz-Garcia et al. (2018b)* and *Schlautman et al. (2015)*** |  |  |
| Berry Color | *BCOLOR* | Digital mean of berry color. |
| Berry Color Variance | *BCOLORVAR* | Digital variance of berry color. |
| September Brix | *BRIX_SEP* | Percent soluble solids for September. |
| October Brix | *BRIX_OCT* | Percent soluble solids for October. |
| September Mean Fruit Mass | *MFM_SEP* | Mean fruit mass for September. |
| October Mean Fruit Mass | *MFM_OCT* | Mean fruit mass for October. |
| October Titratable Acidity | *TA_OCT* | Titratable acidity for October. |
| September Total Anthocyanins | *TACY_SEP* | Total Anthocyanins for September. |
| October Total Anthocyanins | *TACY_OCT* | Total Anthocyanins for October. |
| ***Schlautman et al. (2015)*** |  |  |
| Mean Fruit Mass  ($\frac{g}{berry}$) | *MFM* | Mean fruit mass on 100 randomly sampled berries. |
| Total Yield ($\frac{g}{{ft}^{2}}$) | *TY* | Total yield. |
| Biennial Bearing Index $\left\vert100\times\frac{\Delta TY_{successive}}{\sum TY_{successive}} \right\vert$ | *BBITY* | Biennial Bearing Index of total yield.  $\Delta TY_{successive}$ is the difference in *TY* between successive years, and $\sum TY_{successive}$ is sum of *TY* in successive years. |
| ^a^Bold italic text indicates the study of origin. Entries between parentheses indicate the units of measurement. | | |

Supplementary Table S3. List of categorical traits and their mapped numeric values. Generally, numeric values were chosen such that larger values represented more desirable breeding attributes.

| **Trait** | **Categorical Value** | **Numeric Value** |
| --- | --- | --- |
| Calyx Lobe Form | open (strongly folded back) | 0 |
|  | medium | 1 |
|  | closed (folded over calyx) | 2 |
| Calyx Lobe Size | large | 0 |
|  | medium | 1 |
|  | small | 2 |
| Rebud | no | 0 |
|  | yes | 1 |
| Berry Skin Bloom Level | shiny | 1 |
|  | moderate | 2 |
|  | heavy (waxy) | 3 |

Supplementary Table S4. Comprehensive list of significant QTL, including pairwise interactive QTL, for cranberry (*Vaccinium macrocarpon* Ait*.*) population *CNJ02*. Table fields are as follows:

- *method:* r/QTL package (RRID:SCR_009085) function used to map QTL.
- *model:* Mixed model used to generate BLUPs used as inputs to r/QTL package. Can be one of *2011*, *2012*, *2013*, or *all-years*.
- *trait:* Abbreviated trait name.
- *chr:* Chromosome number for QTL.
- *position:* Genetic position (cM) for QTL.
- *chr2:* Chromosome number for pairwise interaction QTL. *NA* if QTL is additive.
- *position2:* Genetic position (cM) for pairwise interaction QTL. *NA* if QTL is additive.
- *nearest_marker:* Nearest marker on genetic map to QTL.
- *qtl_lod:*QTL LOD score.
- *marker_variance:* PVE of QTL.
- *qtl_pvalue:* p-value of QTL under 1000 permutation.
- *model_variance:* PVE of all QTL found for this trait and model.
- *interval_left:* Smaller genetic position (cM) where LOD profile intersects with 1.5 × LOD below LOD of QTL position.
- *interval:* Width (cM) of interval between *interval_left* and *interval_right*.
- *interval_right:* Larger genetic position (cM) where LOD profile intersects with 1.5 × LOD below LOD of QTL position.

Supplementary Table 5. Comprehensive list of effect sizes for additive QTL in cranberry (*Vaccinium macrocarpon* Ait*.*) population *CNJ02*.

- *method:* r/QTL package (RRID:SCR_009085) function used to map QTL.
- *model:* Mixed model used to generate BLUPs used as inputs to r/QTL package. Can be one of *2011*, *2012*, *2013*, or *all-years*.
- *trait:* Abbreviated trait name.
- *chr:* Chromosome number for QTL.
- *position:* Genetic position (cM) for QTL.
- *genotype:* One of four possible genotypes derived from four-way cross – AC,AD,BC,BD
- *effect_mean:* Mean effect size of QTL for given genotype.
- *effect_se:* Standard error of effect size of QTL for given genotype.
- *AvB:* Maternal effect size - (AC+AD)-(BC+BD).
- *CvD:* Paternal effect size - (AC+BC)–(AD+BD.
- *Int:* Interaction effect size - (AC+BD)-(AD+BC).

Supplementary Table 6. Comprehensive list of significant QTL, including pairwise interactive QTL, for cranberry (*Vaccinium macrocarpon* Ait*.*) population *CNJ04*. Table fields are as follows:

- *method:* r/QTL package (RRID:SCR_009085) function used to map QTL.
- *model:* Mixed model used to generate BLUPs used as inputs to r/QTL package. Can be one of *2011*, *2012*, *2014*, or *all-years*.
- *trait:* Abbreviated trait name.
- *chr:* Chromosome number for QTL.
- *position:* Genetic position (cM) for QTL.
- *chr2:* Chromosome number for pairwise interaction QTL. *NA* if QTL is additive.
- *position2:* Genetic position (cM) for pairwise interaction QTL. *NA* if QTL is additive.
- *nearest_marker:* Nearest marker on genetic map to QTL.
- *qtl_lod:*QTL LOD score.
- *marker_variance:* PVE of QTL.
- *qtl_pvalue:* p-value of QTL under 1000 permutation.
- *model_variance:* PVE of all QTL found for this trait and model.
- *interval_left:* Smaller genetic position (cM) where LOD profile intersects with 1.5 × LOD below LOD of QTL position.
- *interval:* Width (cM) of interval between *interval_left* and *interval_right*.
- *interval_right:* Larger genetic position (cM) where LOD profile intersects with 1.5 × LOD below LOD of QTL position.

Supplementary Table 7. Comprehensive list of effect sizes for additive QTL in cranberry (*Vaccinium macrocarpon* Ait*.*) population *CNJ04*.

- *method:* r/QTL package (RRID:SCR_009085) function used to map QTL.
- *model:* Mixed model used to generate BLUPs used as inputs to r/QTL package. Can be one of *2011*, *2012*, *2014*, or *all-years*.
- *trait:* Abbreviated trait name.
- *chr:* Chromosome number for QTL.
- *position:* Genetic position (cM) for QTL.
- *genotype:* One of four possible genotypes derived from four-way cross – AC,AD,BC,BD
- *effect_mean:* Mean effect size of QTL for given genotype.
- *effect_se:* Standard error of effect size of QTL for given genotype.
- *AvB:* Maternal effect size - (AC+AD)-(BC+BD).
- *CvD:* Paternal effect size - (AC+BC)–(AD+BD.
- *Int:* Interaction effect size - (AC+BD)-(AD+BC).

Supplementary Table 8. Inter-trait Pearson correlation coefficients for cranberry (*Vaccinium macrocarpon* Ait*.*) population *CNJ02*.

Supplementary Table 9. Inter-trait Pearson correlation coefficients for cranberry (*Vaccinium macrocarpon* Ait*.*) population *CNJ04*.
